# Multiple entrained oscillator model of food anticipatory circadian rhythms

**DOI:** 10.1038/s41598-022-13242-w

**Published:** 2022-06-03

**Authors:** Christian C. Petersen, Federico Cao, Adam R. Stinchcombe, Ralph E. Mistlberger

**Affiliations:** 1 Department of Psychology, Simon Fraser University, Burnaby, BC V5A1S6 Canada; 2 Department of Mathematics, University of Toronto, Toronto, ON M5S2E4 Canada

**Keywords:** Circadian mechanisms, Circadian regulation

## Abstract

For many animal species, knowing when to look for food may be as important as knowing where to look. Rats and other species use a feeding-responsive circadian timing mechanism to anticipate, behaviorally and physiologically, a predictable daily feeding opportunity. How this mechanism for anticipating a daily meal accommodates more than one predictable mealtime is unclear. Rats were trained to press a lever for food, and then limited to one or more daily meals at fixed or systematically varying times of day. The rats were able to anticipate up to 4 of 4 daily meals at fixed times of day and two ‘daily’ meals recurring at 24 h and 26 h intervals. When deprived of food, in constant dark, lever pressing recurred for multiple cycles at expected mealtimes, consistent with the periodicity of the prior feeding schedule. Anticipation did not require the suprachiasmatic nucleus circadian pacemaker. The anticipation rhythms could be simulated using a Kuramoto model in which clusters of coupled oscillators entrain to specific mealtimes based on initial phase and intrinsic circadian periodicity. A flexibly coupled system of food-entrainable circadian oscillators endows rats with adaptive plasticity in daily programming of foraging activity.

## Introduction

Pioneering work by Richter^1^ established that rats become active in advance of a daily feeding opportunity in an environment otherwise lacking time of day cues. Anticipation of a predictable but unsignalled daily mealtime (hereafter ‘food-anticipatory activity’, FAA) suggested control of behavior by an internal clock-like mechanism, a concept counter to the behaviorist agenda ascendant at that time. Subsequent analyses of FAA expressed by rats fed once daily revealed canonical properties of an entrained, circadian (~ 24 h) clock-controlled rhythm, including (1) ‘circadian limits’ (robust FAA emerges only in response to feeding cycles in the circadian range)^2–5^, (2) ‘persistence in constant conditions’ (FAA rhythms continue to cycle, i.e., ‘free-run’, across multiple meal omission tests)^4,6–9^, and (3) ‘transient cycles’ following a shift of mealtime (FAA rhythms realign by a series of shifts, rather than a single reset)^10,11^. These properties support a view of FAA as the active phase of a rest-activity rhythm generated by circadian oscillators entrained by a daily meal (hereafter, ‘food-entrainable oscillators’, FEOs)^12–14^. The earliest studies used neurologically intact rats, but circadian properties of food anticipatory rhythms proved to be robust in rats sustaining complete ablation of the suprachiasmatic nucleus (SCN), the site of light-entrainable circadian oscillators indispensable for entrainment to LD cycles^4,7^. FEOs hypothesized to drive FAA must therefore reside in other brain or body parts, although where remains unresolved^15,16^.

The FEO model is complicated by evidence that rats and mice can readily anticipate more than one daily meal^6,7,17,18^. Foundational work by Stephan^19–21^ suggested that SCN-ablated rats with running wheels could anticipate two, but not three daily meals provided at fixed times of day, or, within narrow limits, at systematically varying times within the circadian range (so-called ‘T-cycle’ experiments, where T is the period of the feeding schedule, e.g., 1 meal every 24 h, and a second meal every 25 h). This was interpreted as evidence that FAA is regulated by two FEOs, or groups of FEOs, that can uncouple and entrain independently to one of two daily mealtimes.

The empirical basis for a dual-FEO account of FAA is limited in several important respects. In the 3-meal studies, FAA was measured using running wheels, which may impose metabolic demands that limit expression of FAA^21^. The amount of food consumed at each mealtime, which determines whether FAA will emerge, was assumed to be equivalent but was not controlled or measured. In the two-meal T-cycle experiments, food deprivation tests revealed one, but not two separate bouts of persisting FAA^19,20^. This may be because the expected mealtimes on the food deprivation days were too closely spaced.

We designed experiments to address the limitations in the evidence for a dual-FEO model of meal timing in rats. Our objectives were to (1) test the ability of rats to anticipate more than 2 daily meals, (2) determine whether anticipation of multiple daily meals persists after SCN lesions, and (3) most importantly, assess whether two or more daily bouts of FAA exhibit properties consistent with control by two or more independently entrained circadian oscillators. We then developed a mathematical model, after Kuramoto^22^, to explore the computational plausibility of multiple entrained oscillators as a mechanism for anticipating multiple daily feeding opportunities.

## Results

### Rats readily anticipate three daily meals

**Figure 1:**
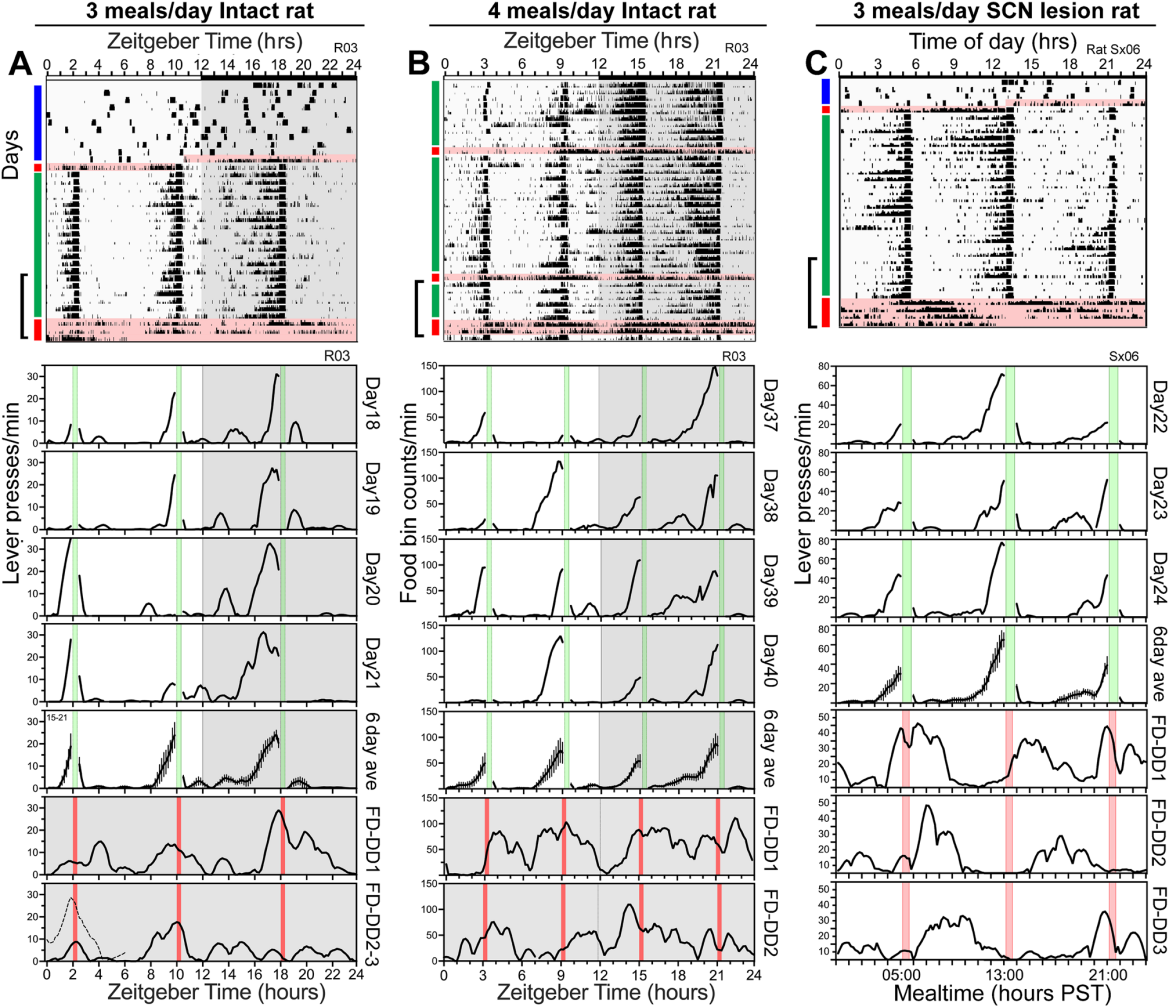
Rats anticipate 3 or 4 daily meals. (**A**) Upper panel: Actogram depicting lever pressing for food in a rat (R03) with food available ad-libitum for 10 days (indicated by blue vertical bar to the upper left of the actogram), followed by 1 day of total food deprivation (pink shading, red vertical bar), 21 days of restricted feeding (green vertical bar; lever pressing reinforced for 30 min starting at ZT2, 10 and 18), and 60 h of total food deprivation (pink shading, red bar). Time of day (10 min bins) is plotted left to right and consecutive days are aligned vertically. Black bars represent time bins with activity. The height of each bar represents relative amount of activity (quintiles). Lower panels: Waveforms of lever pressing for rat R03 on days 18–21 of the 3-meal schedule, and during 60 h of food deprivation in constant dark (FD-DD). Mealtimes are denoted by green vertical bars during scheduled feeding (lever presses yield 20 mg food pellets) and by red vertical bars when lever presses were not reinforced (FD-DD). The fifth panel in the column is the average of the last 6 days of scheduled feeding prior to total food deprivation. Data were smoothed using a 40-min running average. (**B**) Upper panel: Actogram of operant nose-poking for food in rat R03 with 4 daily 15–30 min feeding opportunities, at ZT3, 9, 15, and 21. Lower panels: Waveforms of operant nose-poking for rat R03 on days 37–40 of the 4-meal schedule, and during the 2 days of total food deprivation (FD-DD). The fifth panel is the average of the last 6 days of scheduled feeding prior to the 2-day food deprivation. (**C**) Upper panel: Actogram of lever pressing in a representative SCN-ablated rat (Sx06), fed at 05:00 h, 13:00 h, and 21:00 h local time each day in constant dim light. Lower panels: Waveforms of lever pressing on days 22–24 of the 3-meal schedule, and during the 3 days of total food deprivation. Panel 4 is the average of the 6 days of scheduled mealtimes prior to total food deprivation.

**Figure 2:**
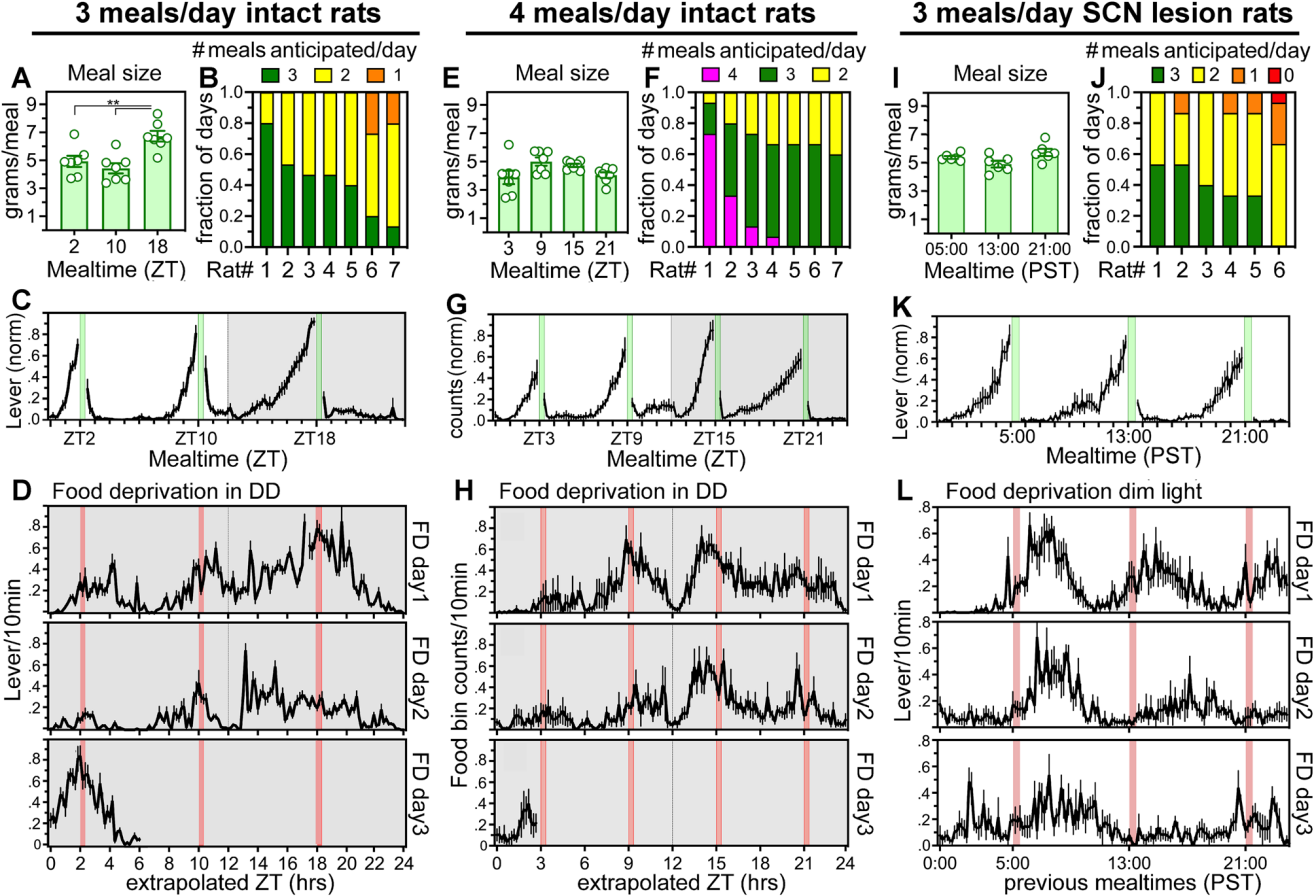
Group mean food intake and food anticipatory activity in rats provided 3 or 4 daily meals. (**A**–**D**) Intact rats on 3-meal schedule. (**E**–**H**) Intact rats on 4-meal schedule. (**I**–**L**) SCN-ablated rats on 3-meal schedule. (**A**, **E**, **I**) Group mean (± SEM) grams food consumed at each mealtime. Significant differences between mealtimes, *p < .001. (**B**, **F**, **J**) Fraction of food restriction days that each rat anticipated 1, 2, 3 or 4 meals (number of daily meals anticipated color coded; red = 0, orange = 1, yellow = 2, green = 3, fuscia = 4). (**C**, **K**) Group mean (± SEM) activity waveforms of lever counts/10 min during the last week of restricted feeding. Mealtimes are denoted by vertical green bars. Operant responses during mealtime were excluded before data normalization and averaging across rats. (**G**) Group mean (± SEM) activity waveforms of operant nose pokes/10 min during the last week of restricted feeding. (**D**, **H**, **L**) Group mean (± SEM) activity waveforms during the food deprivation days in constant dark (**D**, **H**) or constant dim light. (**L**) Food deprivation days 1–3 are aligned vertically. Expected mealtimes denoted by vertical red bars.

Rats housed with running wheels have been reported to anticipate two but not three daily meals^21^. To determine whether this apparent two meal limit reflects a property of the timing system, or is a limitation of the behavioral assay, 7 rats (Group 1) were housed without wheels and trained to press a lever for food (20 mg pellets) in their home cage. FAA expressed in operant behavior shows high precision and temporal specificity compared to wheel running and general locomotor activity^23^. This should in principle facilitate detection of timing behavior when mealtimes are closely spaced, while avoiding excessive energy expenditure in wheel running. After training and 10 days of on-demand feeding, food reward was restricted to three 30-min feeding opportunities each day, beginning at Zeitgeber Times (ZT) 2, 10 and 18 (where ZT is defined relative to the LD cycle, and ZT0 is lights-on, by convention; Fig. 1a). Mealtimes were unsignalled, and therefore lever presses were required to determine food availability. Rats unable to predict mealtimes would need to sample the lever around the clock to find food. Total 24 h food intake was 41 ± 6% (mean ± SD) of ad libitum intake on the first day of the 3-meal schedule, increased to 70% on day 2, and stabilized at 72 ± 5% across the next 3 weeks. Body weights taken at weekly intervals increased by 1.0 ± 0.3% per week. On average, the rats consumed more pellets at the ZT18 (nocturnal) mealtime compared to the ZT2 and ZT10 mealtimes (*F*_6,12_ = 13.7, *p* < 0.0001, last 10 days; Fig. 2a). All of the rats ate some food at all feeding opportunities (Supplementary Fig. S1a).

Food anticipatory lever pressing emerged within the first few meals in all 7 rats (e.g., Fig. 1a; Supplementary Figs. S2a, S3). Importantly, and contrary to earlier studies that used rats housed with wheels, all rats displayed anticipatory activity to all three meals on at least some days, with individual differences in the percent of days on which 1, 2 or 3 bouts of FAA were evident (Figs. 1a, 2b). The rate of lever pressing began to accelerate 2–3 h prior to mealtime and increased monotonically from very low baseline levels to a peak at each mealtime (Figs. 1a, 2c). The amount of lever pressing during the 2 h prior to each meal, expressed as a ratio relative to total intermeal lever pressing, was significantly elevated relative to ratios calculated for the week prior to restricted feeding (*p* < 0.05, Bonferroni-corrected paired t-tests; supplementary Fig. S4a).

### Meal timing persists during extended food deprivation tests

Circadian oscillators have the intrinsic capacity to cycle independently of environmental time cues. Food availability could be anticipated using non-oscillatory timers that function like hourglass clocks or stop-watches. These devices require external input to time successive intervals. To determine whether anticipation of one or more of three daily meals might involve non-oscillatory interval timers, the rats were food deprived for 60 h (7 consecutive mealtimes) in constant dark (DD). If anticipation relies on interval timers reset by each meal or by LD transitions, then it should fail when meals are omitted in DD. Previous studies have shown that rats fed twice daily in the light period continue to become active at scheduled mealtimes during 2- or 3-day food deprivation tests^17^. Similar results were obtained here; rats fed three daily meals showed increased unreinforced lever pressing at most of the mealtimes during food deprivation (Figs. 1a, 2d; supplementary Figs. S2a, S3). The amount of unreinforced lever pressing was greater around the ZT18 mealtime, which corresponds to the middle of the biological night (active phase) in nocturnal rats (note that during food deprivation tests in DD, there is no zeitgeber, and thus no zeitgeber time; ZT notation is used here only for ease of communication).

### Rats can anticipate four daily meals

To further challenge the ability of rats to anticipate multiple daily meals, the rats received food ad libitum for 10 days and were trained to obtain food pellets by inspecting the food-bin, thereby breaking an infrared photobeam, which actuated the pellet dispenser to release a single pellet. This is a natural exploratory behavior which could be a more sensitive assay of meal timing. Following response acquisition and 7 days of on-demand feeding, the rats were food deprived for 24 h and then restricted to three daily meals of 30 min duration, at ZT9, 15, and 21. After 3 days, a fourth daily meal was added at ZT3.

A daily rhythm of food intake was evident during the first week, with most rats showing lower food intake at the ZT3 and ZT9 mealtimes, and consistently high intake at the ZT15 and ZT21 nocturnal mealtimes. On day 15 all 4 meals were skipped (no food intake). On resumption of scheduled feeding, the duration of the ZT15 and ZT21 mealtimes was reduced to 20 min for one week, and to 15 min thereafter. Reducing food intake at those mealtimes served to increase food bin inspections during the ZT3 and ZT9 mealtimes, resulting in equivalent intake at the 4 mealtimes over the last 10 days of the schedule (*F*_3,18_ = 2.192, *p* = 0.123; Fig. 2e).

Anticipatory food-bin inspections emerged within the first few days in all of the rats. Inspection of individual actograms and average waveforms confirmed anticipation of all four meals on many days in 4 of 7 rats (e.g., Figs. 1b, 2f; supplementary Figs. S2b, S5). Three rats failed to anticipate the ZT3 meal and were less likely to eat at this mealtime (Supplementary Fig. S1b) but did anticipate the other 3 meals on most days. Individual and group average waveforms were strikingly similar to the average waves for lever pressing (e.g., Figs. 1b, 2g; supplementary Fig. S5). At the group level, FAA ratios for each mealtime were significant relative to baseline ratios (*p* < 0.05, Bonferroni-corrected paired t tests; supplementary Fig. S4b).

Food bin inspections were unreinforced for 24 h in LD on day 34 of restricted feeding, and for 48 h in DD on days 40–41. All of the rats showed peaks of food-bin activity at most of the expected mealtimes on those days, with food searching weakest at the ZT3 mealtime (e.g., Fig. 1b, 2h; supplementary Fig. S2b, S5). Peaks of food searching behavior at expected mealtimes during total food deprivation rule out non-oscillatory interval timing processes in multiple meal anticipation.

### Anticipation of 3 daily meals does not require the suprachiasmatic nucleus (SCN)

SCN ablation eliminates circadian activity rhythms in rats with free access to food but does not prevent emergence of food anticipatory rhythms by a daily feeding opportunity. To confirm that the SCN is also not required for anticipation of 3 daily meals, 6 rats (Group 2) received bilateral radiofrequency lesions directed at the SCN. Following recovery from surgery, the rats were provided food ad libitum in constant dim light for at least 21 days to assess circadian rhythmicity using motion sensors. All of the rats were arrhythmic in the circadian range, and post-mortem inspection of Nissl-stained sections through the hypothalamus confirmed complete SCN ablations in each case (supplementary Fig. S6a, b).

The SCN lesioned rats were then trained to lever press for food pellets and provided on-demand food access for 10 days. The rats were food deprived for 24 h and then restricted to three 30 min meals per day at 8 h intervals. By the second week of food restriction, average meal size and the probability of eating were equivalent at the three mealtimes (Fig. 2i; supplementary Fig. S1c). Body weights increased by 2.0 ± 1.2% per ~ 10 day interval. All rats showed FAA to each of the three mealtimes (e.g., Fig. 1c; supplementary Figs. S7a, S8). The amount of FAA prior to each mealtime varied across days but, importantly, 5 of 6 rats showed FAA at 3 or more consecutive mealtimes on many days (Fig. 2j). Waveforms for individual rats and the group average were remarkably similar to those from intact rats (Fig. 2k). The rats were then food deprived for 3.5 days (10 mealtimes). Four of 6 rats showed bouts of lever pressing concentrated at each of the missed mealtimes on the first day, with these bouts tending to delay on the second and third deprivation days (e.g., Figs. 1c, 2l; supplementary Figs. S7a, S8). Two rats showed peaks of activity at 2 of 3 mealtimes during food deprivation.

### Single meal omission tests unmask low amplitude food anticipation

**Figure 3:**
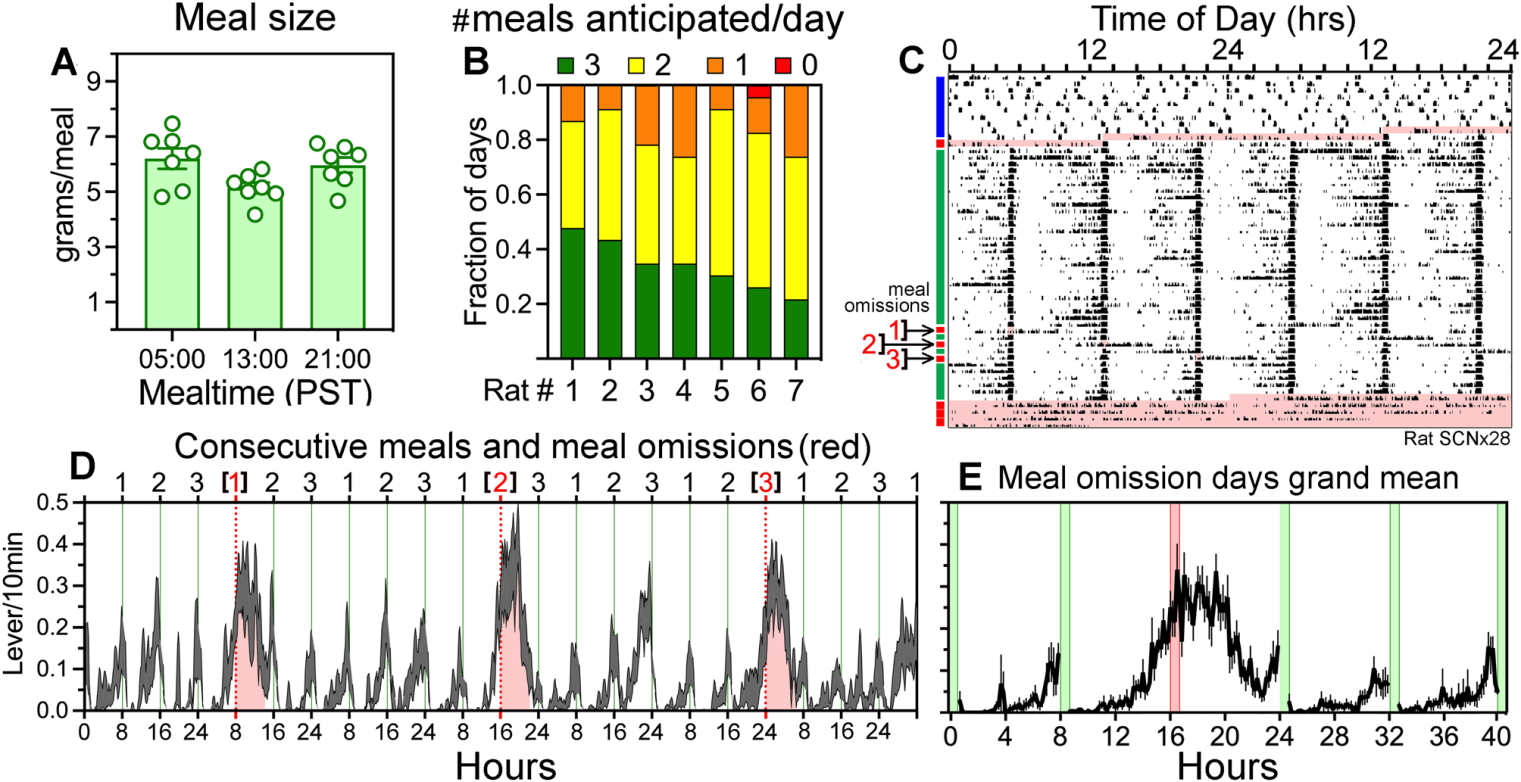
Meal omission tests reveal robust timing of 3 daily meals (**A**) Food intake (grams) at each mealtime (group mean ± SEM). (**B**) Fraction of food restriction days that each rat anticipated 1, 2, or 3 meals (number of meals color coded; red = 0, orange = 1, yellow = 2, green = 3). (**C**) Double-plotted actogram of lever pressing in a representative rat with a SCN lesion estimated to be 95% complete (for histology, see supplementary Fig. S9b). Food was available ad-libitum for 10 days and then restricted to 3 daily mealtimes for 40 days. On days 29, 31 and 33 (denoted by arrows on the left of the actogram) lever pressing was not reinforced during one of the scheduled mealtimes. Pink shading denotes full days of food deprivation. (**D**) Group mean waveform of lever pressing during the week of single meal omission tests. Thin vertical lines indicate scheduled mealtimes. Pink shading denotes unreinforced lever pressing associated with each of the three meal omissions. (**E**) Grand mean (± SEM) of lever pressing during all meal omission test days, generated by aligning and averaging the meal omission days for each rat, and then for the group. Green vertical bars denote mealtimes, and the red vertical bar denotes the omitted meal.

FAA provides evidence of meal timing, but the absence of FAA on a given day is ambiguous, as this could be due to a low hunger level or to variability in the entrained phase of FEOs putatively driving FAA to that mealtime. In either case, withholding food reward (meal omission test) should reveal a peak of operant responding beginning before, during or soon after a regularly scheduled mealtime. To explore this possibility, a second group of rats (N = 7, Group 3) received SCN lesions and were maintained in dim light with food available ad libitum to assess rhythmicity using motion sensors. These rats showed some evidence of activity rhythms in the circadian range (supplementary Fig. S9a), and post-mortem inspection of Nissl-stained sections confirmed partial lesions, ranging from 0 to 95% complete (supplementary Fig. S9b). Nonetheless, when housed in the operant chambers with food reward available on-demand, circadian organization of lever pressing was absent (e.g., Fig. 3c).

When restricted to 3 daily meals, food intake and the probability of eating was equivalent at the three mealtimes (Fig. 3a; supplementary Fig. S1d). All 7 rats showed anticipatory lever pressing to all 3 meals on at least some of the days, but as in previous groups, no rat showed FAA to all 3 meals on every day (Fig. 3b, c). On week 5 of restricted feeding, 3 separate meal omission tests were conducted, at 6 meal intervals. During each of the meal omission tests, all 7 rats exhibited a robust bout of lever pressing at the expected mealtime, regardless of the amount of FAA prior to the mealtime (Fig. 3c,d; supplementary Fig. S10). Averaging across the three separate meal omission tests revealed a Gaussian distribution of unreinforced lever pressing, with counts reaching a peak near the end of the expected 30-min mealtime, plateauing for ~ 4 h, declining and then rising again 1–2 h prior to the next scheduled meal (Fig. 3d, e). Meal omission tests confirmed robust meal timing, even in cases where lever pressing was low or absent prior to a scheduled meal.

### Rats anticipate 2 meals presented at different intervals in the circadian range

The most compelling evidence for a multiple entrained oscillator model of FAA are two reports that rats can anticipate two daily meals with different periodicities in the circadian range^20,21^. In those studies, food deprivation tests could not confirm persistence of distinct bouts of FAA associated with each feeding schedule, which is critical to the dual-FEO interpretation. To address this gap in the evidence, a second group of 7 intact rats (Group 4) were trained to lever press for food reward and provided on-demand reward for 7 days. Food reward was withheld for 24 h and then restricted to a 2 h mealtime at ZT6 for 15 days. The period (T) of the feeding schedule was lengthened to 25 h (T25) for 7 days and to 26 h (T26) for 9 days. A second daily feeding opportunity was then provided at ZT17 (T24) for the next 24 days, after which lever pressing went unreinforced for 72 h. During the concurrent T24/T26 feeding schedules, meal duration was reduced to 1 h/meal.

**Figure 4:**
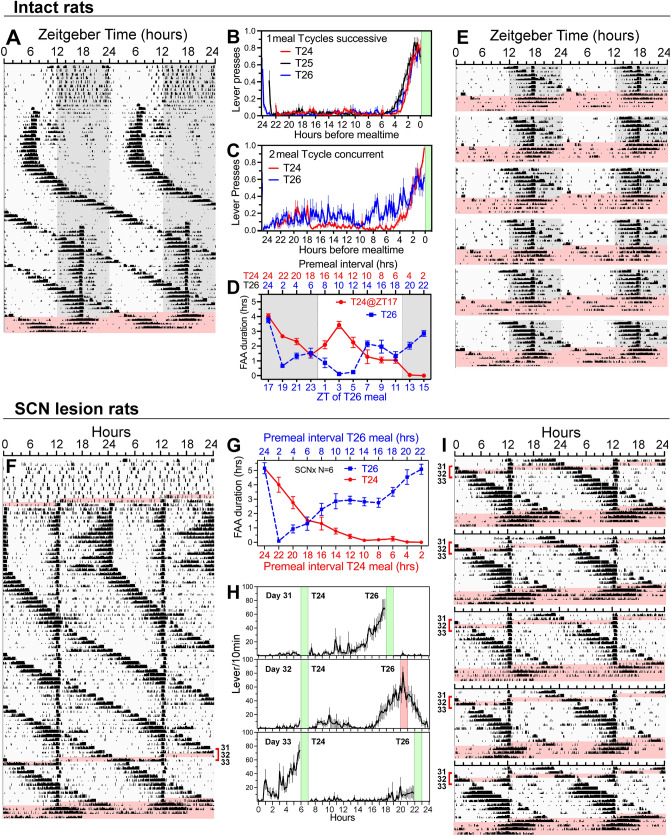
Rats anticipate two daily meals recurring with different circadian periodicities. (**A**) Double plotted actogram of lever pressing for food in an intact rat. Lever pressing reward was provided on-demand for 10 days, then restricted to a 2 h mealtime at ZT6 for 15 days (T24). The feeding cycle was lengthened to 25 h (T25) for 7 days, to 26 h (T26) for 9 days, and then a second meal was provided each day at ZT17 (concurrent T24–T26 schedule). (**B**) Group average (± SEM) waveforms illustrating food anticipatory lever pressing during the single meal T24 (red curve), T25 (black) and T26 (blue) feeding schedules. Data are aligned with mealtime. (**C**) Group mean lever pressing during the concurrent T24 and T26 feeding schedules (**D**) Duration of food anticipatory activity bouts preceding the concurrent T24 and T26 feeding opportunities, plotted as a function of the ZT of the T26 meal. The fasting interval before each mealtime is indicated in hours along the horizontal axis at the top of the graph. (**E**) Actograms for the other 6 rats in this group, showing the last 8 days of the concurrent T24 and T26h feeding schedules, and the 3 days of food deprivation (pink shading). (**F**) Actogram of lever pressing in a representative rat with a complete SCN ablation, provided two daily feeding opportunities first at 12 h intervals, and then at 24 h and 26 h intervals concurrently. Pink shading denotes food deprivation days. (**G**) Duration of food anticipatory activity bouts preceding the concurrent T24 and T26 feeding opportunities, plotted as a function of the premeal fasting interval. (**H**) Group mean average waveforms of lever pressing on days 31–33 of the concurrent T24 and T26 schedules. The T26 meal was skipped on day 32. (**I**) Actograms of lever pressing from the other 5 SCN-ablated rats in this group, showing the meal omission day (day 32), the last 9 days of the concurrent T24 and T26 schedules, and the 3 day food deprivation test (pink shading).

When food was available once daily at ZT6, all rats exhibited a prominent bout of lever pressing beginning ~ 2 h prior to mealtime (e.g., Fig. 4a; Supplementary Fig. S11). When the feeding interval was increased to 25 h and then to 26 h, FAA continued to track mealtime. FAA during the single meal T cycles was compared by averaging the data modulo-T (Fig. 4b). FAA onset was earlier at the longer feeding intervals, a result consistent with principles of oscillator entrainment (note that the mealtimes used to generate the three waveforms did not occur at the same times of day, which may have contributed to waveform differences).

When a second daily feeding opportunity was introduced at ZT17, FAA to this meal emerged within a few days, while FAA to the T26 mealtime became more variable (e.g., Fig. 4a). Averaging the data modulo-24 h across 13 days (to remove the T26 FAA) and modulo-26 h across 12 days (to remove the T24 FAA) reveals a slightly longer duration and lower amplitude peak of FAA prior to the T26 meal (Fig. 4c). FAA bout duration shows systematic variability related to the intermeal interval and time of day relative to the LD cycle (Fig. 4d). FAA duration to both meals increased with the amount of time since the last meal. FAA to the T26 meal was shortest when those meals occurred early in the light period. Despite this variability, visual inspection of the actograms clearly reveals many days on which lever pressing anticipated both meals (Fig. 4a,e).

### Food deprivation reveals period matching of FAA rhythm and feeding schedule

After 24 h days of concurrent T24 and T26 feeding schedules, lever pressing was unreinforced for 90 h in constant dark. All rats exhibited a prominent bout of lever pressing centered during the hours previously associated with the T24 mealtime (i.e., ZT17-18; Fig. 4a,e). A bout of lever pressing was also evident at a time predicted by the T26 schedule for at least 1 cycle, and in 4 of 7 cases for multiple cycles. Notably, in these cases, the activity bouts previously associated with the T24 and T26 schedules recurred with periodicities approximating 24 h and 26 h, respectively. To quantify periodicity, activity onsets and ends identified using the Clocklab algorithm were used to estimate the center of each FAA bout. A regression line was then fit to this phase estimate. The group mean periodicity was 23.84 ± 0.34 h (range 23.18–24.25 h) for the T24 FAA bout, and 26.12 ± 0.64 h (range 25.33–27.22 h) for the T26 FAA bout (Supplementary Fig. S13). This period matching is reminiscent of ‘aftereffects’ on the period of SCN-driven rhythms free-running in DD that are evident following entrainment to non-24 h LD cycles in the circadian range^24^.

### Anticipation of two meals with different periods does not require the SCN

To confirm that anticipation of 2 daily meals recurring at 24 h and 26 h intervals does not require the SCN, the rats with complete SCN lesions (Group 2) were provided food ad libitum for 10 days, food deprived for 24 h and then restricted to two daily 1-h meals at 12 h intervals for 15 days. One of the mealtimes was then delayed by 1 h/day (T25) for 17 days and then by 2 h/day (T26) for 41 days. The T26 meal was omitted on day 32, when it would have occurred 10 h before the T24 meal. The schedule culminated with a 72-h food deprivation test.

When food reward was available twice daily at 12 h intervals, all of the SCN-ablated rats showed anticipatory lever pressing to both meals on most days (e.g., Fig. 4f; Supplementary Fig. S12). When one meal was delayed by 1 h and then 2 h each day, anticipatory lever pressing was evident to both meals on some days, but as with intact rats, the amount of FAA to the two feeding schedules varied with the preceding intermeal interval and showed a strong reciprocal relation (Fig. 4g). FAA was greatest when the preceding intermeal interval was 24 h (both meals at the same time), creating a ‘beat frequency’ of 12 days. FAA was generally weaker to the T24 schedule but was enhanced on the day that the preceding T26 meal was omitted (Fig. 4f,h,i). When lever pressing was unreinforced for 72 h, all rats exhibited two main bouts of activity, with one more prominent bout recurring with a group mean period of 23.9 ± 0.41 h (range 23.32–24.38 h) and a second bout recurring with a period of 26.62 h ± 0.37 h (range 26.07–27.0 h) (Fig. 4f,i; Supplementary Fig. S13). In several cases (Fig. 4i), the bout previously associated with the T24 feeding appeared to advance as the ~ 26 h bout delayed. A 2-way ANOVA confirmed a significant main effect of feeding cycle on FAA periodicity in DD, in both the intact and SCN-ablated groups (*F*_1,11_ = 138.0, *p* < 0.0001; 88% of variation), but no main effect of group (*F*_1,11_ = 3.96, *p* = 0.072; 1.1% of variation).

### Anticipation of multiple daily meals can be simulated with a Kuramoto oscillator model

**Figure 5:**
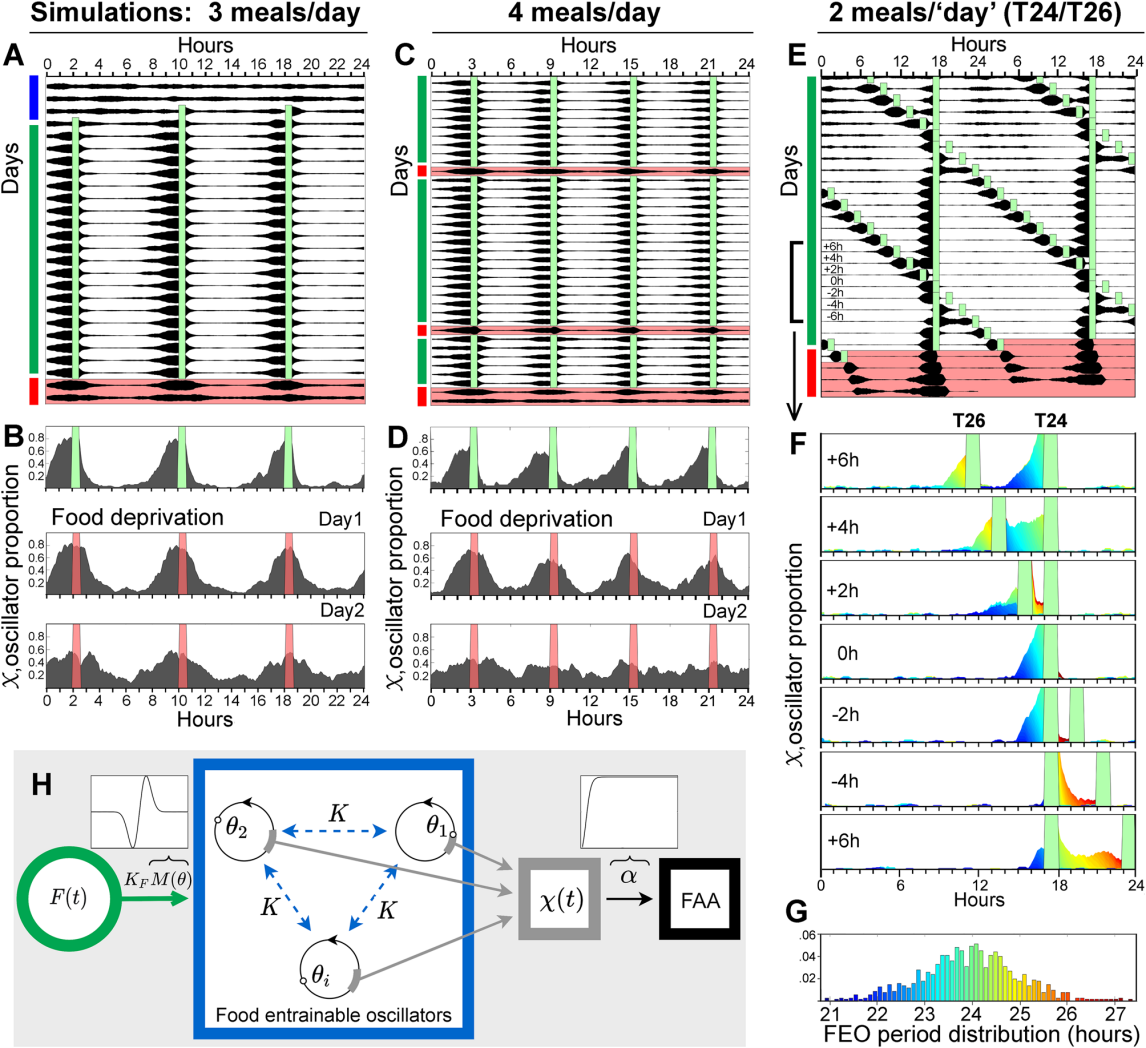
A Kuramoto model simulates food anticipatory activity rhythms. Actograms and running waveforms showing predicted activity $\mathrm{\alpha }\left(\upchi \right)$ of multiple FEOs in response to 3 daily meals (**A**, **B**) 4 daily meals (**C**, **D**), and 2 daily meals provided at different periodicities (24 h and 26 h, **E**). Plotting format as in Fig. 1a. Green bars denote mealtimes. Red bars and pink shading denote expected mealtimes during a 2 or 3-day food deprivation test. (**F**) Waveforms illustrating the phase FEO oscillators colored by intrinsic periods during concurrent T24 and T26 meal schedules. Oscillators with periods > 24 h entrain to the 26 h meal and oscillators with periods < 24 h to the 24 h meal. (**G**) Frequency distribution and color-coding of FEO periodicities within the 21–28 h range. (**H**) Diagram of components of the quantitative model (see "Methods" for details).

Based on these data we postulate that FAA represents the output of a population of coupled FEOs that entrain to specific mealtimes based on initial phase proximity and intrinsic period. We formulate and interrogate this concept using a Kuramoto model in which FEOs are represented by 1000 coupled phase oscillators sampled from a population with intrinsic periodicities normally distributed within the circadian range (Fig. 5g,h; see "Methods" section for details). For simplification, and given the similarities between the intact and SCN-ablated rats, this iteration of the model leaves out interactions with SCN oscillators.

Simulations of the 3-meal schedule demonstrate excellent agreement with experimental results (Fig. 5a). The model generates activity that rises monotonically a few hours before expected mealtimes, peaks at mealtime, and decreases sharply after the food is removed (Fig. 5b). Simulations of the 4-meal schedule also yield good qualitative agreement with observed behavior, although discrepancies are evident during the intermeal intervals, where the model produces slightly more activity than observed in the experiment (Fig. 5c,d). The amount of activity between meals (including FAA) is likely influenced by non-circadian factors, such as hunger and satiety, which may differ in the 3- and 4-meal schedules, given differences in meal duration and deprivation interval. Future extensions of the model will explore homeostatic and other potential modulatory factors, including SCN output, environmental light, characteristics of the food (e.g., palatability), and variations in body temperature.

Simulations of the total food deprivation tests are also in general agreement with the main features of the data (Fig. 5b,d). The model generates bouts of activity that recur for several cycles at the appropriate time of day. The bouts widen as the peak level declines. This may be explained by considering the food stimulus function $F(t)$, which controls the drive from food intake onto FEO phase. Due to its restoring nature, $F(t)$ attracts and synchronizes oscillators rapidly to $\theta = 0$. If food is not provided, the oscillators begin to disperse naturally, owing to their Gaussian distribution of periods and weak coupling. Thus, the waveform appears nearly symmetric about the expected mealtime as it gradually damps across days. This corresponds with the experimental evidence.

Simulations of FAA observed in the 2-meal T cycle experiments (concurrent 24 h and 26 h meals) are illustrated in Fig. 5e. Remarkably, the model successfully reproduces two essential features of the data, including anticipation of both meals, and period matching of FAA bouts to the prior feeding schedules during the extended food deprivation test.

The frequency distribution of FEO period lengths used in the simulation is illustrated in Fig. 5g. Color coding was employed to visualize how FEOs with different periodicities entrain preferentially to matching T cycles during a week of days when the T26 meal crossed over the T24 meal (Fig. 5f). This plotting format clearly shows the sorting of oscillators by intrinsic period, with slower oscillators (τ > 24 h) aligning with the longer T cycle and retaining this alignment after the cross over.

Persistence of the 2 rhythms in the food deprivation simulation appears to be more robust compared to the 3- and 4-meal food deprivation simulations. The conditions are not strictly comparable, but if damping rate is related to period heterogeneity within the cluster of FEOs driving an FAA bout, and if the clusters that entrain to T24 and T26 schedules are more homogeneous than they would be if both schedules were T24, then a slower rate of damping is predictable.

## Discussion

By making food availability contingent on time of day and instrumental behavior, we have shown that rats can anticipate 3 or 4 daily meals at fixed times of day, and 2 daily meals recurring at different periodicities in the circadian range. Importantly, during meal omission tests, peaks of anticipation recurred at expected mealtimes based on the time of day or periodicity of each feeding event. Persisting rhythms of instrumental food seeking behavior, with periodicities approximately matching the previous feeding cycle, provide strong evidence for a multiple entrained oscillator model of food anticipatory activity. The remarkable similarity of these rhythms in the intact and SCN-ablated rats indicates that the SCN pacemaker is not required to time multiple bouts of anticipation, although it may modulate FEO inputs (e.g., by affecting meal size) and/or FEO outputs (activity level). Successful simulation of the anticipation rhythms with a Kuramoto model lends computational plausibility to the multiple FEO concept.

Although all but one of 13 rats (7 intact and 6 SCN-ablated) tested on the 3-meal schedule showed anticipation of 3 or more consecutive meals across one or more consecutive days, none anticipated all meals on every day. In rats fed 2 daily meals at concurrent 24 h and 26 h periodicities, food anticipation was strongly modulated by time since last feeding, and in the intact rats, also by time of day relative to the LD cycle. On some days, there was little activity prior to one or the other meal. In these cases, low or zero amplitude FAA most likely reflects processes downstream from FEOs responsible for timing FAA. This interpretation is based on the unmasking of FAA by meal omission tests. Strikingly, in the SCN-ablated rats, when one scheduled T26 mealtime was skipped, unreinforced lever pressing exhibited a high amplitude Gaussian distribution, peaking shortly after the expected mealtime. FAA to the next scheduled T24 meal was then markedly increased over low levels evident on the previous 2 days. Clearly, the rats were timing the T24 meal, even when they exhibited little or no FAA to that meal on recent days. Meal timing is further indicated by the fact that the rats never failed to lever press before the end of the T24 feeding opportunity, unless (in 2 rats) that meal was preceded within 4 h by the previous T26 feeding opportunity.

Four of 7 intact rats provided 4 daily feeding opportunities exhibited FAA prior to each of 4 daily meals on at least some days. The other 3 rats showed consistent FAA primarily to the two nocturnal mealtimes, and on some days failed to inspect the food bins during the ZT3 mealtime. Food intake is required for anticipation to emerge, and it is possible that if mealtimes had been signalled by a light or tone, all rats would have eaten at every mealtime, and anticipation of all 4 meals might then have emerged. Unsignalled mealtimes present the greater challenge, and by using this more stringent procedure, we may underestimate the capacity of rats to consistently anticipate 4 daily meals.

By contrast with individual rats, anticipation viewed at the group level was robust at all mealtimes, as illustrated by the group mean waveforms. Rats live in colonies and exhibit social learning and social transmission of foraging information^25,26^. If there is social transmission of foraging opportunities linked to time of day, then rats in colonies may be able to anticipate more daily feeding opportunities, and with greater consistency and precision, than could any solitary rat. Entrainment of circadian oscillators by food may in turn reinforce social synchrony within colonies.

The Gaussian distribution of unreinforced lever pressing evident during meal omission tests in the present study is reminiscent of response distributions generated by rats and mice working for food under fixed, short interval reinforcement schedules (seconds to minutes range; e.g.^27^). In other respects, food anticipatory rhythms observed here and in other studies of circadian feeding schedules are distinct from anticipatory responding to short non-circadian intervals. Most importantly, the anticipatory cycle of responding generated by fixed interval reinforcement schedules in the seconds to minutes range does not persist for more than one cycle when operant responses are not rewarded. FAA rhythms generated by circadian schedules, and by the 2-, 3- and 4-meal schedules used in the present study, persist for multiple cycles during food deprivation tests.

Symmetrical intermeal intervals were used in the 3- and 4-meal studies, as a strategy to minimize variability in the amount of food consumed at different mealtimes. Symmetrical intervals raise the possibility that FAA could be driven by ultradian oscillators with periodicities of 8 h and 6 h, respectively. However, this would not account for anticipation of 2 meals with different periodicities in the circadian range. Also, numerous studies have shown robust anticipation of two daily meals with asymmetrical intermeal intervals (e.g.^17,18^).

A multiple FEO model interprets each bout of FAA as the active phase of an entrained rest-activity cycle driven by a unique cluster of FEOs. In some species, a LD-entrained circadian clock can function as a continuously consulted chronometer, permitting time-compensated sun-compass orientation^28,29^. The capacity to continuously ‘read’ (i.e., discriminate) circadian clock phase is also assumed to underlie the ability of some birds and mammals to learn feeding locations contingent on time of day^30–32^. Discriminating the phase of a single entrained clock could be a basis for anticipating multiple daily meals at fixed times of day, but to account for anticipation of 2 meals with different periodicities, computations of an unlikely degree of complexity would be required (e.g., learn an interval timing rule that the T26 meal will occur 2 h later than it did the day before, *relative to the T24 meal*, and continue to apply the rule even if the T24 meal is omitted, i.e., measure the intervals *relative to the T24 clock* and not the T24 meal). For these reasons, we favor multiple entrained oscillators to explain anticipation and leave open the possibility that outputs from these oscillators can also be encoded in time-place memories. Ultimately, these conceptual models will need to be validated at the biological level.

A multiple FEO model of FAA, whereby anticipation of different daily meals is mediated by different entrained circadian oscillators, is conceptually aligned with the dual pacemaker model of LD entrainment^24,33^. One challenge for any multiple oscillator model is to explain how oscillators entrained to one mealtime avoid interference from meals at other times of day. We present a solution based on heterogeneity of periodicity within a population of coupled circadian oscillators, as in the Kuramoto model^22^. Oscillators within the population entrain to one of 3 or 4 daily meals by a process of sorting based on their initial phase relative to the mealtimes. Oscillators entrain to 24 h or 26 h feeding cycles by sorting based on oscillator period. According to this conception, apparent ‘aftereffects’ of the two feeding cycles on the periodicity of FAA rhythms that persist during extended food deprivation tests reflects the intrinsic properties of oscillators entrained to each meal, rather than a modification of oscillator period by prior entrainment. The results do not rule out the possibility that entrainment can modify FEO periodicity, but the model indicates that plasticity of τ is not required to explain differences in FAA periodicity in the free-running state. Heterogeneity of oscillator period as an adaptive feature of multi-oscillator circadian systems has recently been highlighted^34^, and the results and simulations presented here provide additional support for this conjecture.

The multiple FEO model presented here can account for other characteristics of food-anticipatory rhythms reported elsewhere. Aschoff^35^ noted that FAA induced by feeding rats one meal per day was often accompanied by a bout of activity after mealtime and proposed that the two bouts together represent the active phase (α) of a food-entrained circadian clock. A multiple oscillator model would further propose that activity before and after a daily mealtime would represent α of FEOs with shorter and longer circadian periodicities, respectively. Consistent with this proposal, Luuk et al.^36^ observed that in some mice anticipating a midday meal, when ad-libitum access was reinstated, activity associated with FAA persisted with advancing transients, while a bout of activity at the end of the mealtime phase delayed before merging with nocturnal activity onset. Similarly, Nishide et al.^37^ noted that FAA in mice may be associated with bouts of nocturnal activity that advance or delay toward mealtime, depending on mealtime relative to the LD cycle. This was suggestive of circadian ‘bout’ oscillators, a concept previously invoked to explain the daily structure of rest-activity bouts in free-feeding Syrian hamsters^38,39^. A multiple FEO model would propose that bout oscillators as a population express intrinsic periodicities that are normally distributed within a circadian range. Mice do not tolerate the extended food deprivation tests that are typically needed to fully characterize FAA timing mechanisms, but multiple meal schedules indicate an ability to anticipate 2 or more daily meals, consistent with a multiple FEO model^17,18,40^.

Future extensions of the model will incorporate additional factors thought to influence the timing and magnitude of FAA bouts. Candidate factors include a homeostatic process that modulates the motivation to express appetitive behavior as a function of time since the last meal, a second circadian process that represents the effects of SCN output on FEO timing and rest-activity states, and environmental factors, such as light intensity. These extensions are expected to improve the performance of the model in reproducing essential features of FAA in intact and SCN-ablated rats.

Behavioral studies such as reported here leave unresolved the neural and physiological mechanisms of food-anticipatory rhythms. Efforts to identify cells, genes and signals responsible for generating and entraining food anticipatory rhythms have understandably focused almost exclusively on single daily meal schedules. Feeding schedules with multiple daily mealtimes provide additional experimental contexts for designing critical tests of proposed mechanisms.

## Methods

### Animals

Young adult male Sprague–Dawley rats (crl:SD, N = 27) were obtained at ~ 2 months age (225–250 g) from Charles River Laboratories (Quebec, Canada). The rats were housed individually in operant chambers inside a humidity and temperature controlled (~ 22°) vivarium in a 12:12 light:dark (LD) cycle or constant dim light (~ 60 lx). Diet consisted of 20 mg food pellets (Purina TestDiet Precision Pellets, St Louis, MO; Formula PJPPP, percent of calories by weight: 20.6% protein, 12.7% fat, 66.7% carbohydrates) with water available ad libitum throughout all experiments. Animal health was checked daily when experimental conditions allowed. Cages were cleaned, and rats weighed weekly. All experimental procedures were approved by the University Animal Care Committee at Simon Fraser University (permit 1233p-17) and conformed to the Canadian Council on Animal Care’s Guide to the Care and Use of Experimental Animals and Canada’s Animals for Research Act. The study is reported in accordance with ARRIVE guidelines.

### Apparatus

Operant chambers (N = 7, model ENV-008, Med-Associates, NY) were housed within individual ventilated sound-attenuation cubicles (model ENV-018MD) equipped with white lights (~ 60 lx) and an exhaust fan. The front panels of the operant chambers were outfitted with a retractable lever and a food trough. Food troughs were modified by use of a photobeam to allow for detection of food bin inspections (‘nose-pokes’). Pellets were provided via a 20 mg pellet dispenser positioned outside the chamber and attached to the food trough. Operant chambers were modified to allow for insertion of water bottles and plastic tops were replaced with wire mesh to allow for recording of locomotor activity using overhead infrared motion sensors. Operant responses (lever presses or nose-pokes) and reinforcements were summed and stored in 1-min intervals. Lever operation, reinforcement delivery, and data collection were controlled by a Pentium PC running Med-PC for Windows software (version 4.24; Med-Associates). Activity counts measured by overhead motion sensors were summed and stored in 1-min intervals using the ClockLab data acquisition system (Actimetrics, IL, US).

### Surgery

Rats undergoing surgery for SCN ablation (N = 6, 275–350 g at the time of surgery) were anesthetized using a combination of ketamine (90 mg/kg, IP) and xylazine (15 mg/kg, IP) and pretreated with Metacam (1 mg/kg, SC) and 5 cc lactated ringer solution. Two bilateral radiofrequency lesions (total of four lesions per animal) were aimed at the SCN, using stereotactic coordinates set at 0 mm and 0.5 mm posterior to bregma, ± 0.4 mm lateral, and 9.3 mm ventral to skull, with the head levelled. A direct current was passed for 15 s through epoxy coated insect pins (#01, ~ 0.05 mm tip exposed). Following surgery, anesthesia was reversed using atipamezole (1 mg/kg, SC) and the rats were treated with buprenorphine (0.02 mg/kg, SC) and 5 cc lactated ringer solution. Following three days of post-operative care, the rats were returned to the experimental cages to assess circadian rhythmicity of locomotor activity. Rhythms were evaluated by visual inspection of actograms and by chi-square and Lomb-Scargle periodogram analyses of a 10-day segment during which the rats were not disturbed.

### Histology

Following behavioral testing, rats with SCN-lesions were deeply anesthetized with CO^2^ and perfused through the heart with 0.01 M PBS followed by 4% paraformaldehyde. Brains were extracted and post-fixed overnight in 4% paraformaldehyde and then transferred to 30% sucrose solution for at least 2 days. Frozen sections (50 μ) were cut in the coronal plane through the anterior hypothalamus. Sections were mounted on slides and stained with cresyl violet for light microscopic examination of lesion placement.

## Procedures

### Group 1 (N = 7 intact rats): 3- and 4-meal schedules

#### 3-Meal feeding schedule

Group 1 rats housed in a 12:12 LD cycle were trained to press a lever for a 20 mg food pellet reward using a continuous reinforcement schedule with food obtained only via operant responses. Following response acquisition and 7 days of on-demand (ad libitum) feeding, the rats were food deprived for 24 h and then restricted to three 30-min meals/day presented at 8-h intervals (ZT2, ZT10, and ZT18, where ZT0 is lights-on, by convention). Unreinforced operant responding prior to mealtime was used as a measure of anticipatory activity. Meal durations for each rat were adjusted as necessary to keep meal sizes approximately equal across mealtimes. Following 21 days on the three-meal schedule, Group 1 rats were food deprived in constant dark (DD) for 50 h.

#### 4-Meal feeding schedule

Following 7 days with food available ad libitum, Group 1 rats were trained to ‘nose-poke’ (break a beam over the food cup) for a 20 mg pellet reward. Following response acquisition and another 10 days of on-demand feeding, the rats were food deprived for 24 h and then restricted to four ~ 20-min daily mealtimes presented at 6-h intervals (ZT3, 9, 15, and 21). The rats were maintained on the 4-meal feeding schedule for 40 days. On day 15 a 1-day food deprivation test was conducted in DD. On day 33, a second 1-day food deprivation test was conducted in LD. After day 40, the rats were food deprived in DD for 2 days.

### Group 2 (N = 6 rats with complete SCN lesions): 3-meal schedule and 2-meal T cycle

#### 3-Meal schedule

Following recovery from surgery and confirmation of arrhythmicity, the SCN-ablated rats were trained to lever press for a food pellet reward. Following response acquisition and 10 days of on-demand feeding, the rats were food deprived for 24 h and then provided three 30-min feeding opportunities per day, at 8 h intervals. After 28 days, the rats were food deprived for 86 h (10 mealtimes).

#### 2-Meal T cycle schedule

The rats received food reward on-demand for two weeks, were food deprived for 24 h, and then provided two 1-h feeding opportunities per day, at 12 h intervals, for 15 days. One mealtime was then delayed by 1 h/day for 17 days (T25) and then by 2 h/day for 41 days (T26), while the other mealtime was unchanged (T24). On day 31, no food reward was provided at the T26 mealtime (single meal omission test). After day 41, the rats were food deprived for 3 days.

### Group 3 (N = 7 rats with partial SCN lesions): 3-meal schedule with single meal omission tests

#### 3-Meal schedule

Following recovery from SCN-lesion surgery and assessment of rhythmicity, the rats were trained to lever press for a food pellet reward. Following response acquisition and 10 days of on-demand feeding, the rats were food deprived for 24 h, provided three 30-min feeding opportunities per day, at 8 h intervals, for 40 days. and then food deprived for 86 h (10 mealtimes). On days 29, 31 and 33, no food was provided at one scheduled mealtime.

### Group 4 (N = 7 intact rats): 2-meal T cycle schedule

#### 2-Meal T cycle schedule

Group 4 rats were trained to press a lever for food pellets. Following response acquisition and 10 days of on-demand reward, food was restricted to a one 2 h daily meal at ZT6 (6 h after lights-on). After day 14, mealtime was delayed by 1 h/day (T25) for 7 days and then for 2 h/day (T26) for 9 days. A second meal was then introduced at ZT17 (5 h after lights-off), and both meals were set to 1 h duration. Following 27 days of concurrent T24 and T26 feeding, the rats were food deprived in DD for 90 h.

### Statistical analysis

Group mean values in the text are reported ± SEM. For visual inspection, general cage activity (measured by overhead infrared motion sensors) and operant responses (lever pressing or nose-poking) were summed in 10-min bins and plotted in the standard raster format using Clocklab (Actimetrics, IL, USA). Bivariate plots of activity averaged over days were created using Prism 9.0 (Graphpad Software Inc., La Jolla, CA, USA). Meal anticipation was quantified as the sum of activity occurring in the 1.5 h (4-meal schedule) or 2 h (3-meal schedule) preceding a mealtime as a proportion of either the total amount of daily activity minus feeding activity (in whole-day FAA ratios), or the total amount of activity during the intermeal interval (intermeal FAA ratios). FAA onsets for each mealtime were determined visually using Clocklab software.

To evaluate differences in quantitative measures of anticipation between feeding conditions separate two-tailed repeated-measures analyses of variance (ANOVAs) were conducted, followed with paired t tests with Bonferroni corrections where a significant main effect was found (alpha pre-set to 0.05). All statistics were analyzed using Prism 9.0 and SPSS (Version 19, IBM, Armonk, NY).

### Mathematical model

We propose that a population of FEOs gives rise to food anticipatory behaviour. We model this population with $N$ coupled oscillators as in the Kuramoto model^22^. Each oscillator is described by its phase, ${\uptheta }_{i}\in \left[\mathrm{0,2\pi }\right].$ We assume that each FEO has a period ${T}_{i}$ in the circadian range, and that the distribution of periods within the population is normally distributed with a mean of $\upmu = 24$ h and a standard deviation of $\upsigma = 1$ h. The model is deterministic once the period distribution has been sampled.

The phases change in time according to a coupled system of differential equations

$$\dot{{\uptheta }_{i}}=\frac{2\uppi }{{T}_{i}}+\frac{K}{N}{\sum \limits_{j=1}^{N}}{\text{sin}}\left({\uptheta }_{j}-{\uptheta }_{i}\right)-{K}_{F}\mathrm{F}\left(t\right)\mathrm{M}\left({\uptheta }_{i}\right), \mathrm{i}=1,\ldots ,\mathrm{N},$$

in which $K$ is the coupling strength between the oscillators and ${K}_{F}$ is the strength of the phase modulation exerted by feeding related input. The function $M\left(\uptheta \right)$ represents the phase modulation by food and it is essential that it is local, restoring, and periodic. We assume that $M\left(\uptheta \right)$ is given by a truncated Fourier sine series,

$$M\left(\uptheta \right)={\sum\limits_{j=1}^{8}}{a}_{j}\,\mathrm{sin}\,j\uptheta .$$

Parameter values for the Fourier coefficients ${a}_{j}$ are listed in Table 1. $M\left(\uptheta \right)$ is required to be local for the formation of multiple oscillator clusters, each representing entrainment to one meal among one or more daily meals. It is required to be restoring so that the oscillators may be attracted to a particular mealtime, which we define as phase angle $\uptheta =0$. The requirement for periodicity is clear. Hence, the parameters were selected so that the oscillators are unaffected by the stimulus outside a 6 h interval around a particular mealtime, and within this interval, the drive due to the presence of the meal gradually increases as the oscillators approach $\uptheta = 0$. A plot of $M\left(\uptheta \right)$ is included in the model diagram (Fig. 5h).

**Table 1:** Parameter values used in the model differential equations.

Parameter	Value	Description
$K$	0.001	FEO intercoupling
${K}_{F}$	0.6433	Food input strength
$N$	1000	Number of FEOs
$\upsigma$	1	Period population standard deviation
$\upmu$	24	Period population mean
${\theta }_{*}$	0.2	Phase duration for meal indication
${k}_{1}$	40	Rate of saturation
${k}_{2}$	5	Location of saturation
${a}_{1},\dots ,{a}_{8}$	0.2142, 0.3295, 0.3177, 0.2259, 0.1236, 0.0523, 0.0170, 0.0041	Fourier coefficients of $M\left(\uptheta \right)$

The function F*(t)* represents the food stimulus and is defined using a step function: *F(t)* attains a value of 1 when food is presented and 0 during the absence of food. The primary output of the FEOs is the proportion of the oscillator population near phase zero, $\upchi \left(t\right).$ When an oscillator is near $\uptheta = 0$, i.e., $\uptheta \in \stackrel{\sim }{\uptheta }=\left[-{\uptheta }_{*},0\right],$ the oscillator is signalling a prediction of mealtime to the rat. Specifically,

$$\upchi \left( t \right) = \frac{1}{N}\sum\limits_{{i = 1}}^{N} {1_{{\tilde{\uptheta }}} \left( {\uptheta _{i} (t)} \right)} ,$$

in which $1_{{\tilde{\uptheta }}}$ is the indicator function. The value of ${\uptheta }_{*}$ is selected to correspond to ~ 45 min to give good qualitative agreement with the experimental data.

We propose an instantaneous output of the signalling provided by the FEOs of the Kuramoto model. With the proportion of oscillators in the phase duration for meal indication $\upchi \left(t\right)$, the instantaneous output of this proportion $\mathrm{\alpha }\left(\upchi \right)$ is represented by a sigmoid using the exponential function,

$$\mathrm{\alpha }\left(\upchi \right)=\frac{1}{1-c}\left(\frac{exp\left({k}_{1}\upchi \right)}{exp\left({k}_{1}\upchi \right)+{k}_{2}}-c\right), c = \frac{1}{1+{k}_{2}}.$$

The instantaneous output $\mathrm{\alpha }\left(\upchi \right)$ contains two parameters. The parameter ${k}_{1}$ defines the rate of saturation while the parameter ${k}_{2}$ defines the location of saturation. $c$ shifts the sigmoid so that it passes through the point (0,0) and 1/(1 − c) normalizes the sigmoid so that it attains a maximum value of 1. The model parameters (Table 1) were chosen to best match qualitatively with experimental actograms and activity waveform plots.

The model components are illustrated in Fig. 5h. Food $F\left(t\right)$ acts on the FEOs through ${K}_{F}M\left(\theta \right)$, the oscillators ${\theta }_{i}$ are mutually coupled and the model outputs the raw proportion of these oscillators $\chi \left(t\right)$ within $\stackrel{\sim }{\theta }$, which is then filtered by $\alpha$ to become FAA. Note that the anticipatory behavior observed through the model is not a prescribed process, rather, it is because the FEO periods are distributed about some mean with some standard deviation. As a result, some oscillators possess shorter periods while some possess longer periods than the mean. When there is a sufficient density of oscillators of the specific cluster arriving ahead of mealtime, the rat is signalled that food will become available. In our model, this signal is simply the proportion $\upchi \left(t\right)$, modulated by the instantaneous output $\mathrm{\alpha }$.

The numerical simulations were performed in MATLAB R2020b. For replication purposes, we set a fixed seed in the random number generator, rng(1). The period distributions were constructed using MATLAB’s normrnd function.

The ODEs were solved using ODE45 with a randomly distributed uniform initial condition over $\left[\mathrm{0,2\pi }\right]$. Because the food stimulus is piecewise continuous, we solved over each region of continuity in the ODE solver to avoid a loss of accuracy with ODE45. The absolute and relative tolerance was chosen to be 1e-10.

## Supplementary Information

Supplementary Figures.

## Data Availability

All data are available on reasonable request, directed to the corresponding author, Ralph Mistlberger (mistlber@sfu.ca).
