# Supplementary material for: Multiple entrained oscillator model of food anticipatory circadian rhythms
Source: Sci Rep. 2022 Jun 3;12:9306. doi: 10.1038/s41598-022-13242-w (PMC9166752; doi:10.1038/s41598-022-13242-w)

**Supplementary Figure S1.**

Probability of individual rats eating at each scheduled mealtime, when food reward was freely available (baseline week) and when restricted to 3 or 4 daily meals (last 10 days). **A,B**. Group 1, N=7 intact rats**. C.** Group 2, N=6 rats with complete SCN **ablation. D.** Group 3, N=7 rats with partial SCN lesions.


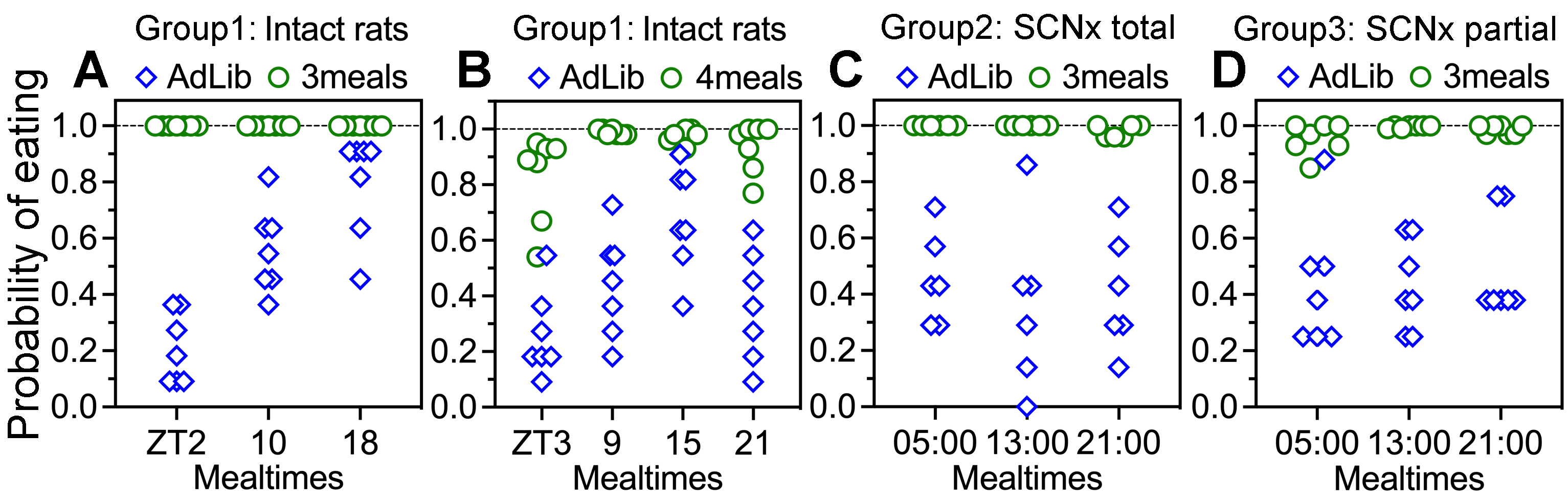


**Supplementary Figure S2.**

Individual actograms from Group 1 rats (R1-R7) provided 3 [A] or 4 [B] daily feeding opportunities, contingent on lever pressing [A] or food bin beam breaks [B]. Mealtimes are denoted by the green shading in the two actograms on the top row of each column. Lights off is denoted by grey shading, and total food deprivation days by pink shading.


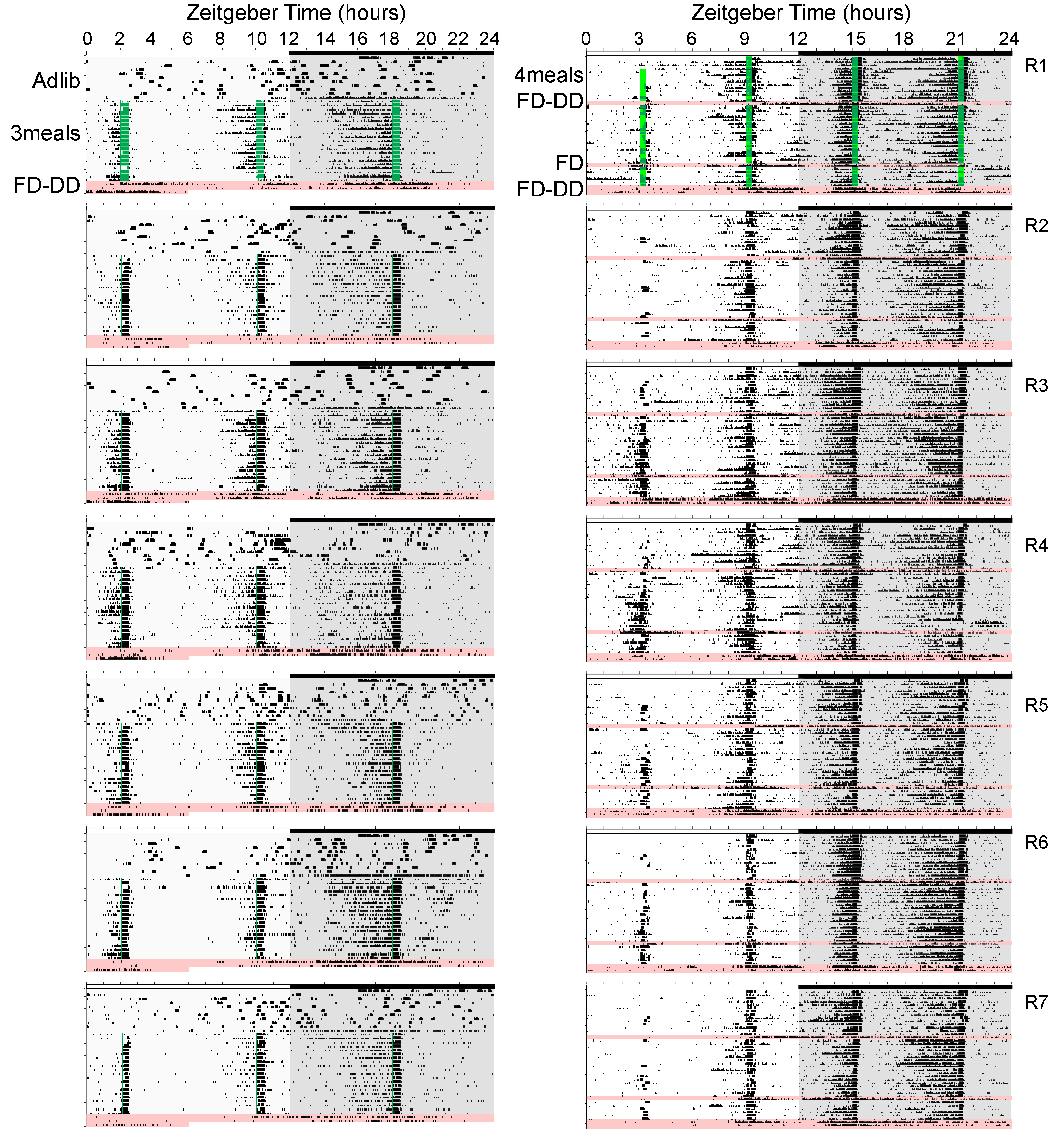


**Supplementary Figure S3.**

Lever pressing activity waveforms of Group 1 rats during the last 7 days of the 3-meal feeding schedule and the 2 days of total food deprivation. Each set of 9 panels represents a different rat. In the last panel in each set, the lighter of the two curves denotes the first 6h of the third day of food deprivation.


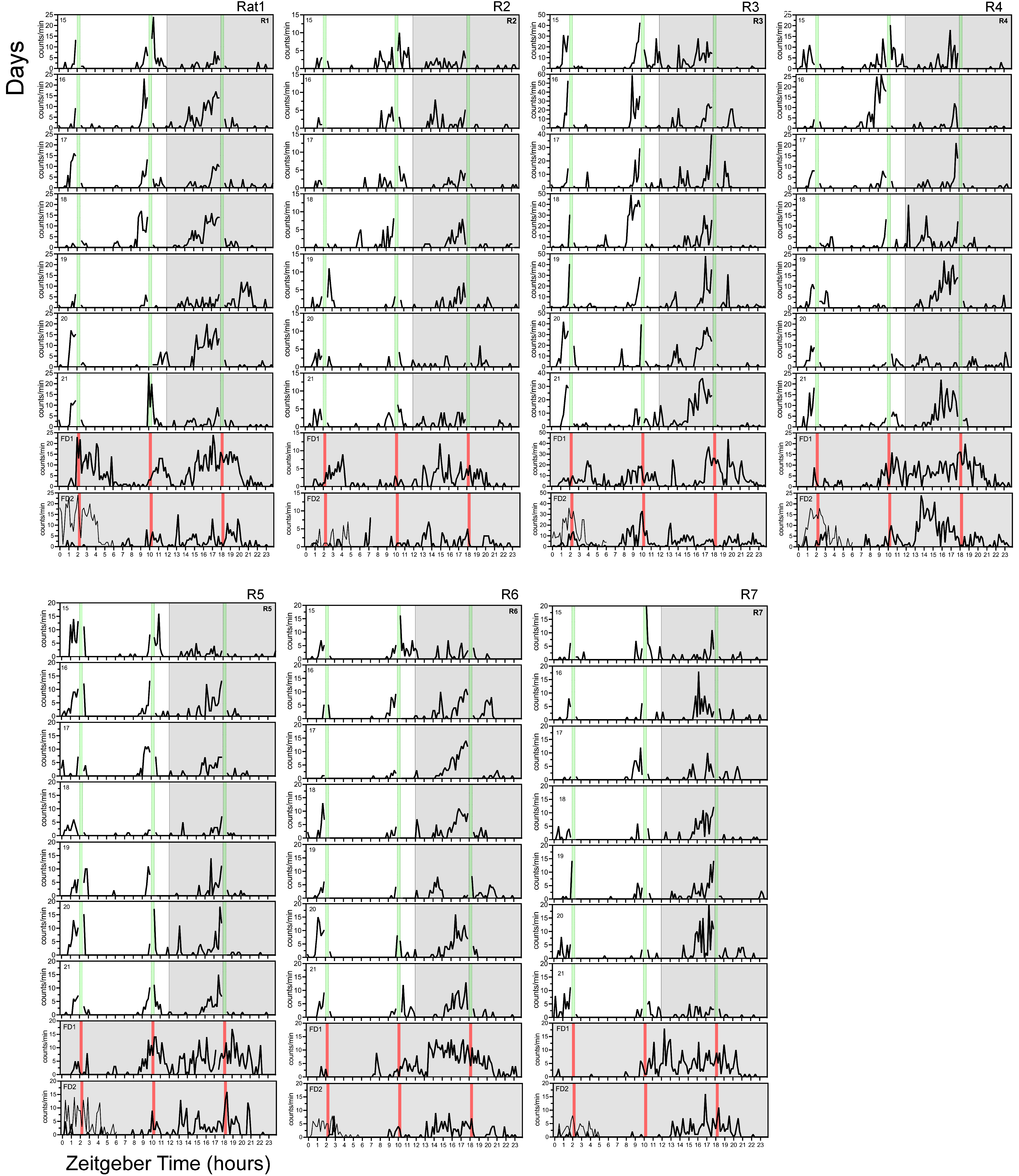


**Supplementary Figure S4.**

Individual and group mean food anticipatory activity ratios for intact rats (Group 1) with [A] 3 or [B] 4 daily feeding opportunities and SCN-ablated rats (Group 2) with [C] 3 feeding opportunities. Ratios were calculated by summing lever [A,C] or food bin beam break [B] counts during the 1.5 h [B] or 2 h [A,C] prior to mealtime, and converting these to a ratio relative to total daily activity, excluding mealtimes. Individual data points represent the means for each rat during the last week of ad-lib food access (blue symbols and bars) and the last week of restricted feeding (green symbols and bars). Group differences significant by post hoc tests following 2-way repeated measures ANOVA. Abbreviations: AL = adlib days, RF = restricted feeding days.


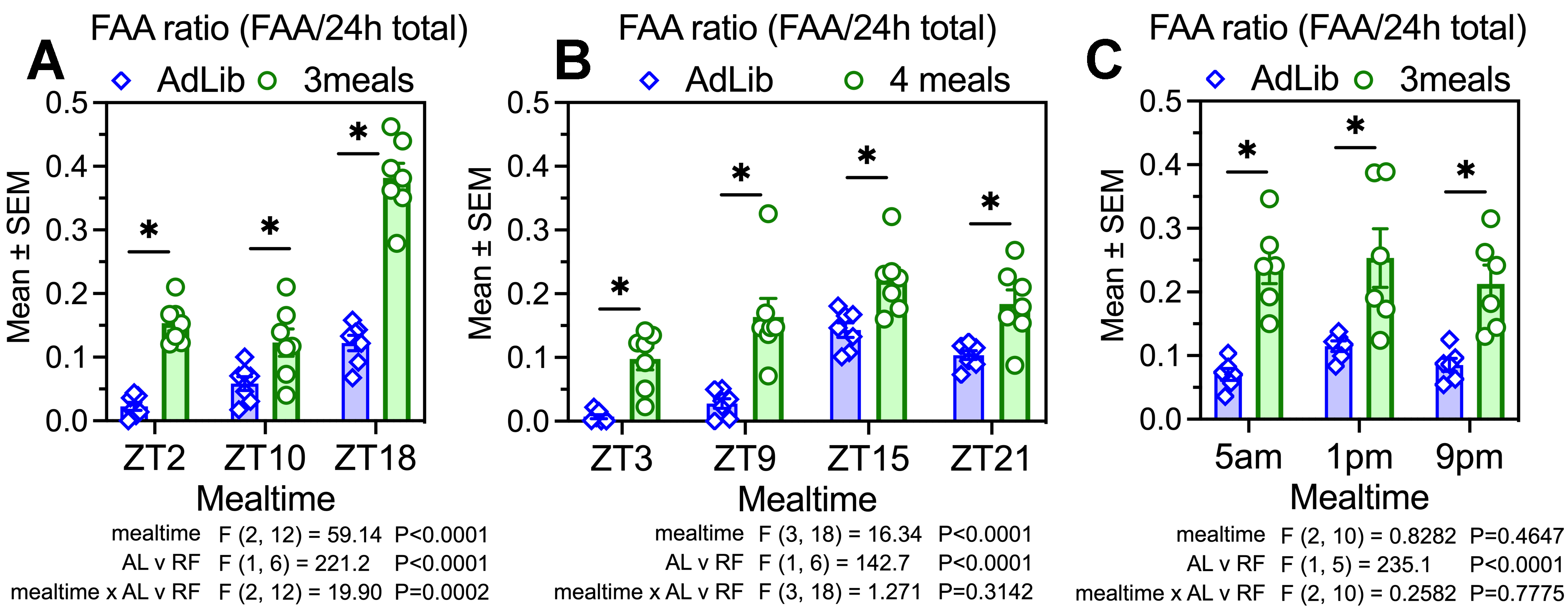


**Supplementary Figure S5.**

Operant food bin activity of individual rats during the last 6 days of the 4-meal feeding schedule and the 2 days of total food deprivation in constant dark. Each set of 8 panels represents a different rat. Mealtimes are denoted by vertical green bars. Lever pressing during mealtimes is omitted. Expected mealtimes during the food deprivation days are denoted by red shading.


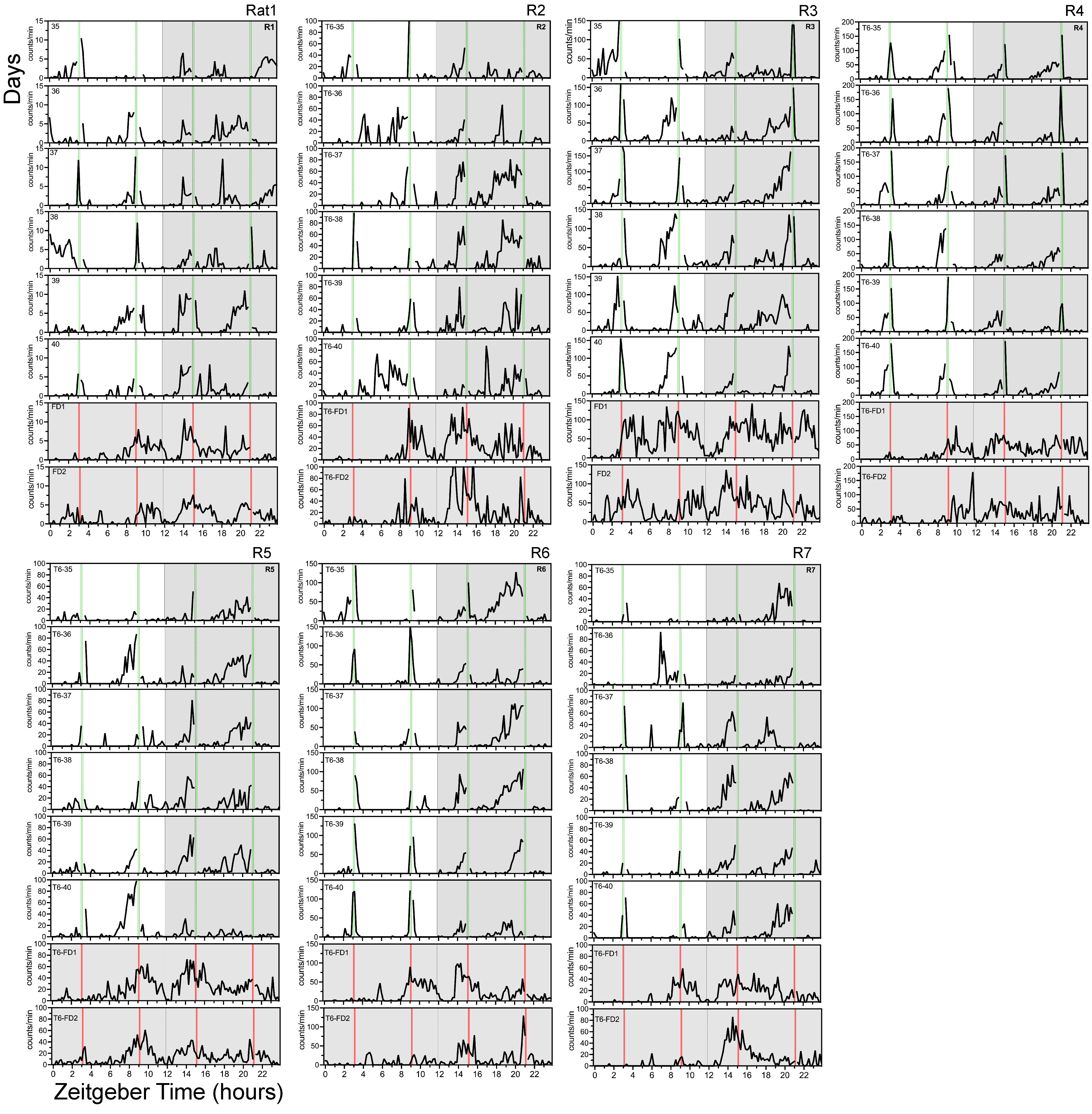


**Supplementary Figure S6.**

Behavioral and histological confirmation of complete SCN ablation. [A] General cage activity measured by motion sensors in 6 rats that received complete SCN lesions (SCNx1,6,7,10,11,12) and one sham lesion rat (sham-19). [B]. Nissl stained brain sections illustrating the SCN lesions in each rat, at 4 levels from just anterior to just posterior to the SCN.


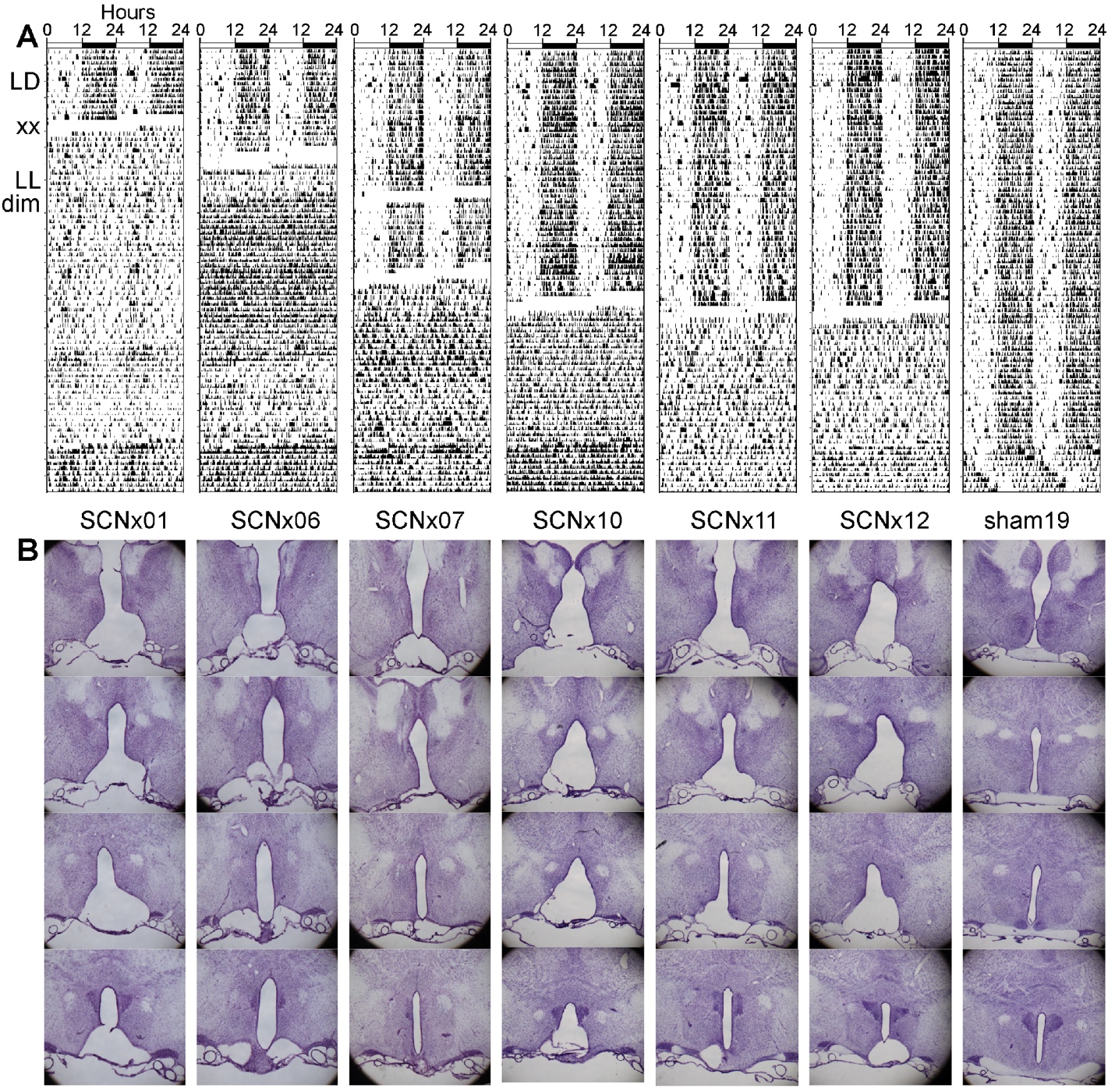


**Supplementary Figure S7.**

Double-plotted actograms of lever pressing activity in rats with SCN lesions provided 3 daily feeding opportunities in constant dim light. A. Rats with complete SCN lesions. [B] Rats with partial lesions. Brackets to the right of the actograms indicate 7 day segment with 3 single meal omission tests. Mealtimes are denoted by green shading in the upper left panel. Food deprivation days are denoted by pink shading.


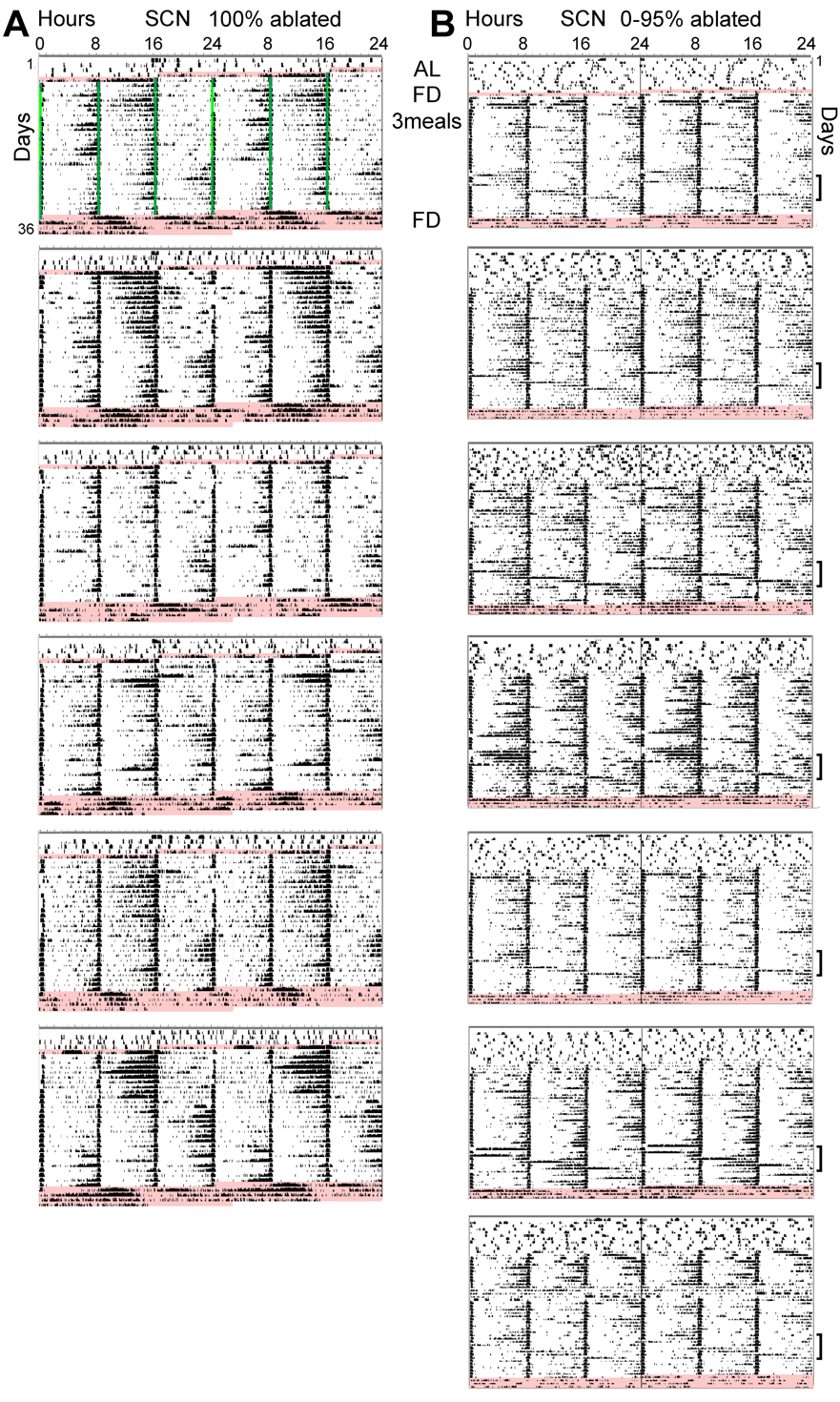


**Supplementary Figure S8.**

Waveforms of lever pressing of individual SCN-ablated rats (Group 2) during the last 7 days of the 3-meal feeding schedule and the 3 days of total food deprivation. Each set of 10 panels represents a different rat. Mealtimes are indicated by vertical green bars, and by vertical red bars on the food deprivation days. Lever presses during scheduled meals are omitted for scaling purposes.


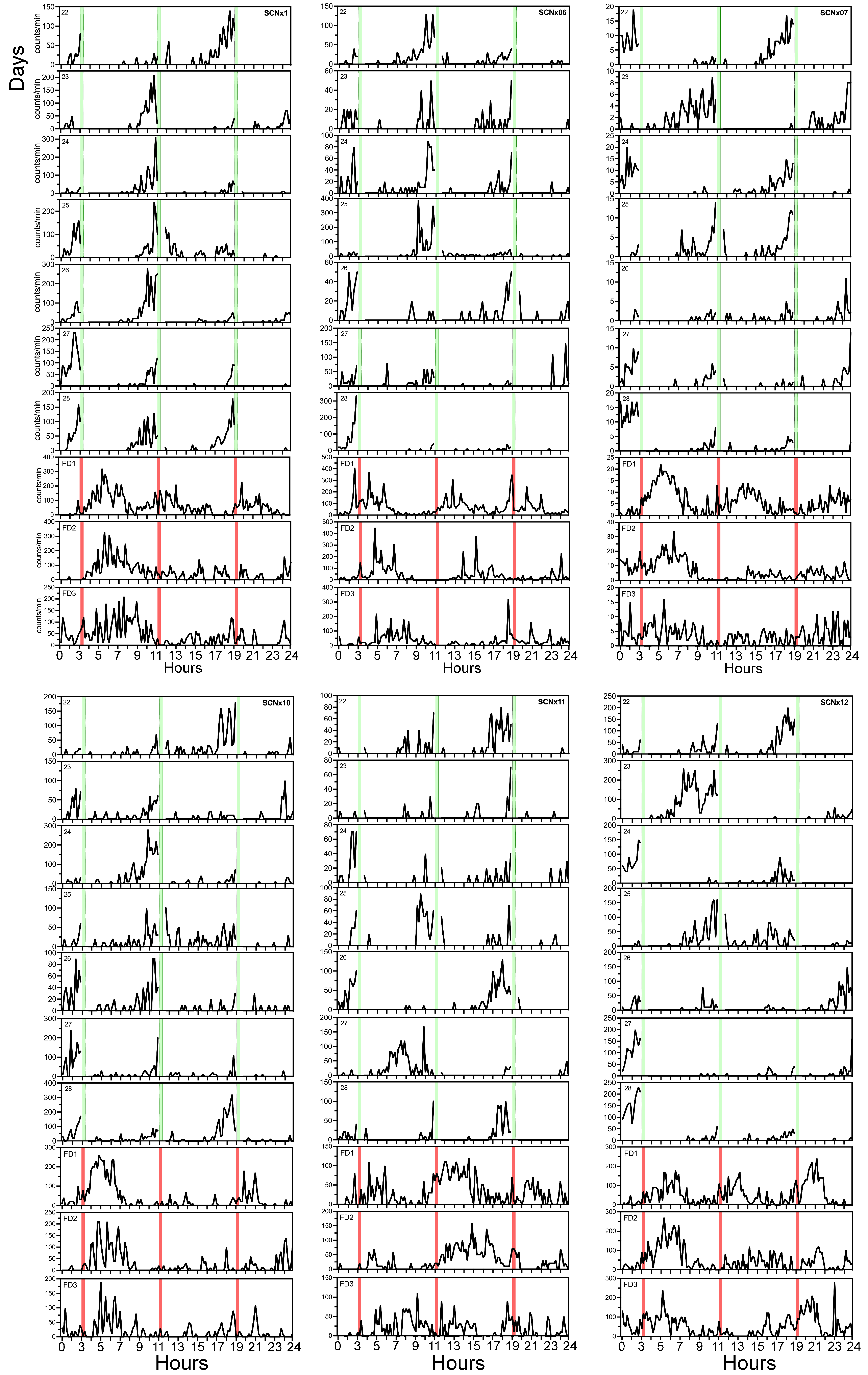


**Supplementary Figure S9.**

Partial SCN lesions. [A] General cage activity measured by motion sensors in 7 rats with partial SCN lesions [B]. Nissl stained brain sections illustrating the SCN lesions in each rat, at 4 levels from just anterior to just posterior to the SCN. Percentages represent the amount of SCN tissue estimated to be ablated.


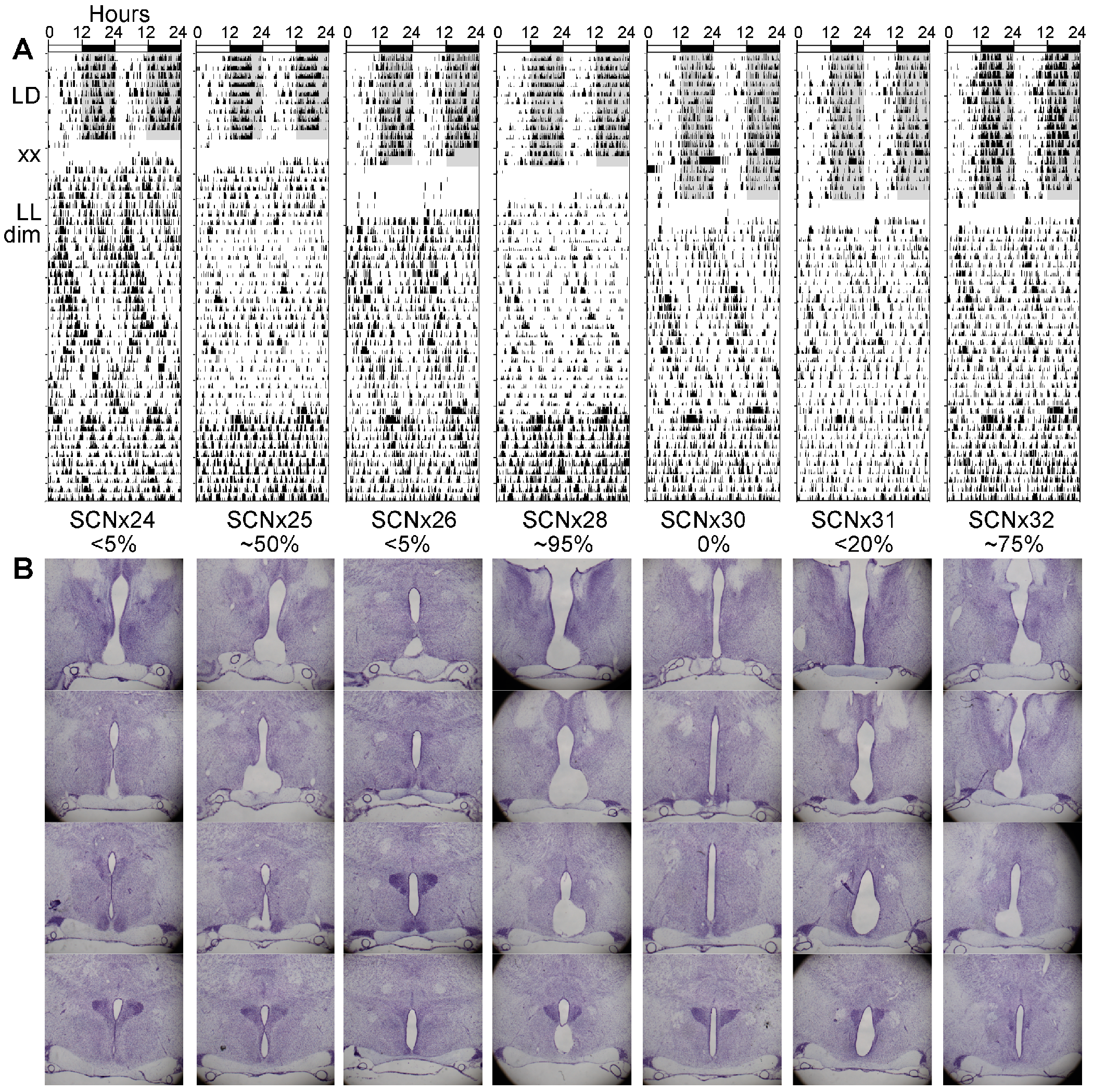


**Supplementary Figure S10.**

Lever pressing of rats with partial SCN lesions (Group 3) on 3-meal schedule, during a week of single meal omission tests. Left column: 7 day running waves of normalized lever pressing counts per 10 min bin for the group (top row) and the 7 individual rats. Right column: Data averaged across food deprivation days, aligned by meal omission day. Pink shading denotes meal omissions. Green vertical bar denotes mealtimes.


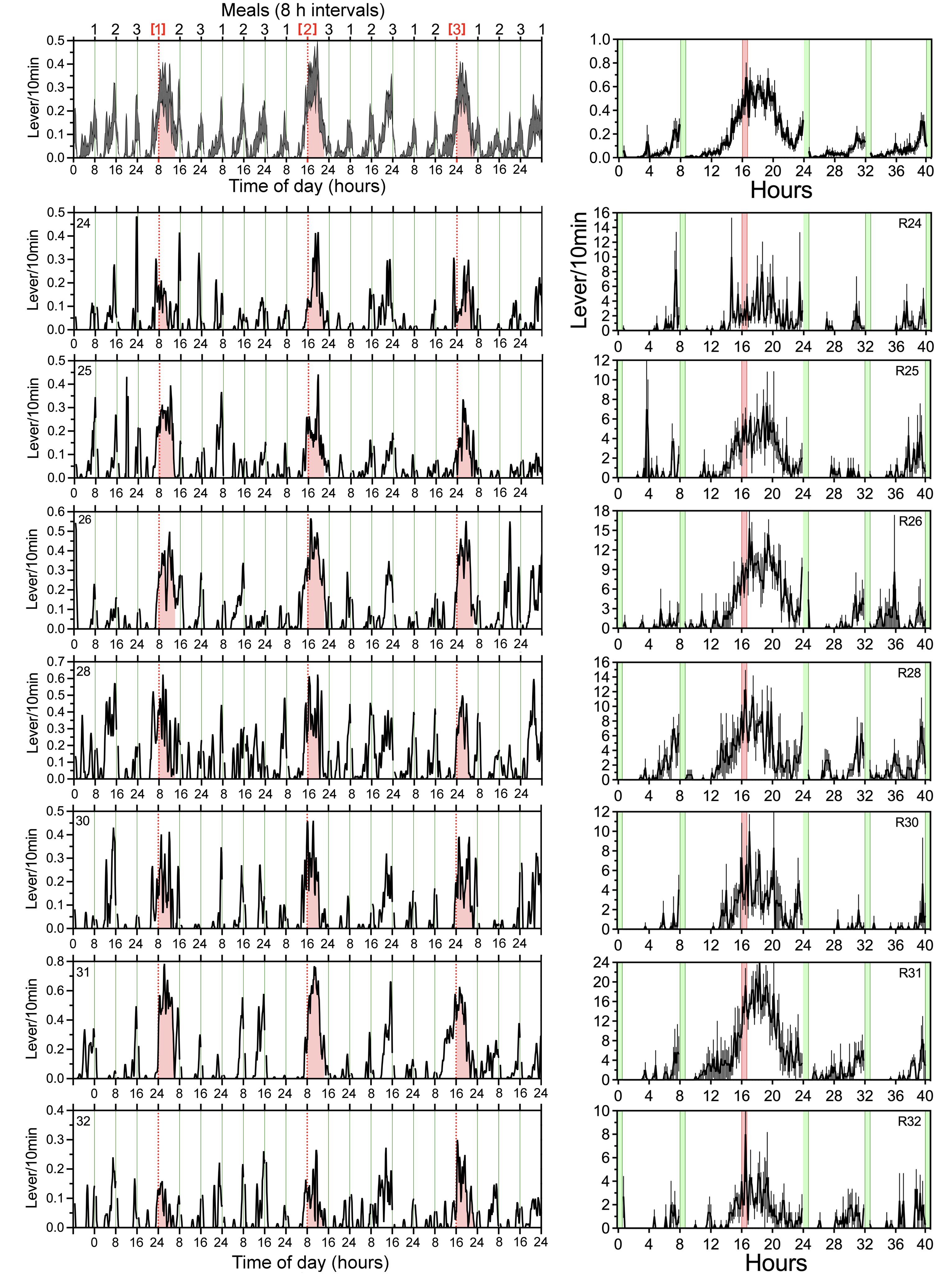


**Supplementary Figure S11.**

Individual actograms of lever pressing from rats provided 1 daily feeding opportunity at intervals of 24, 25 or 26 h and then 2 daily opportunities at concurrent feeding cycle periodicities of 24h and 26h. The blue vertical bar along the left axis denotes food available ad-libitum. The green bar denotes restricted feeding days. The red bar and pink shading denotes food deprivation days.


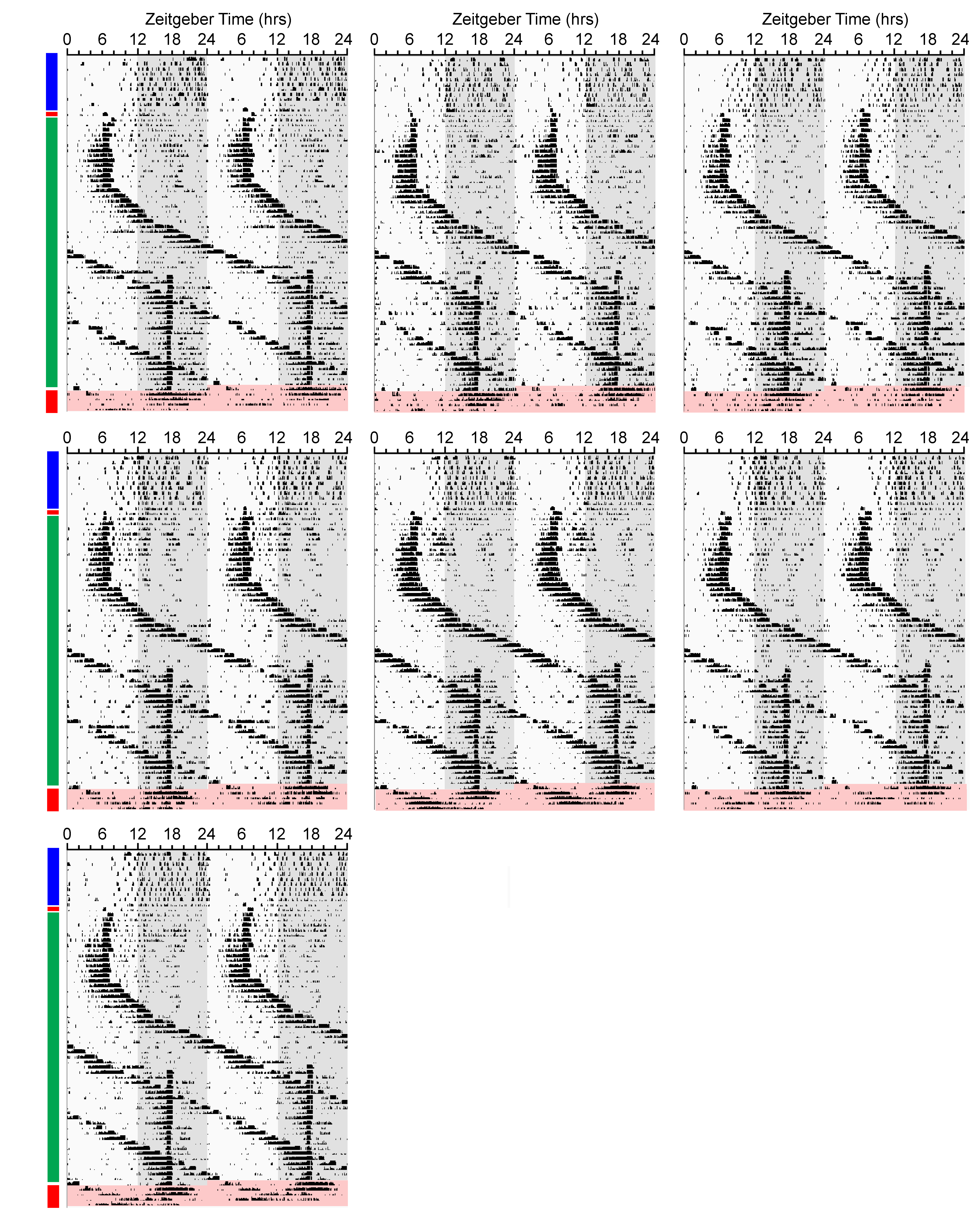


**Supplementary Figure S12.**

Individual actograms of lever pressing from SCN-ablated rats provided 2 daily feeding opportunity at 12h intervals and then 2 daily opportunities at concurrent feeding cycle periodicities of 24h and 26h. The blue vertical bar along the left axis denotes food available ad-libitum. The green bar denotes restricted feeding days. The red bar and pink shading denotes food deprivation days


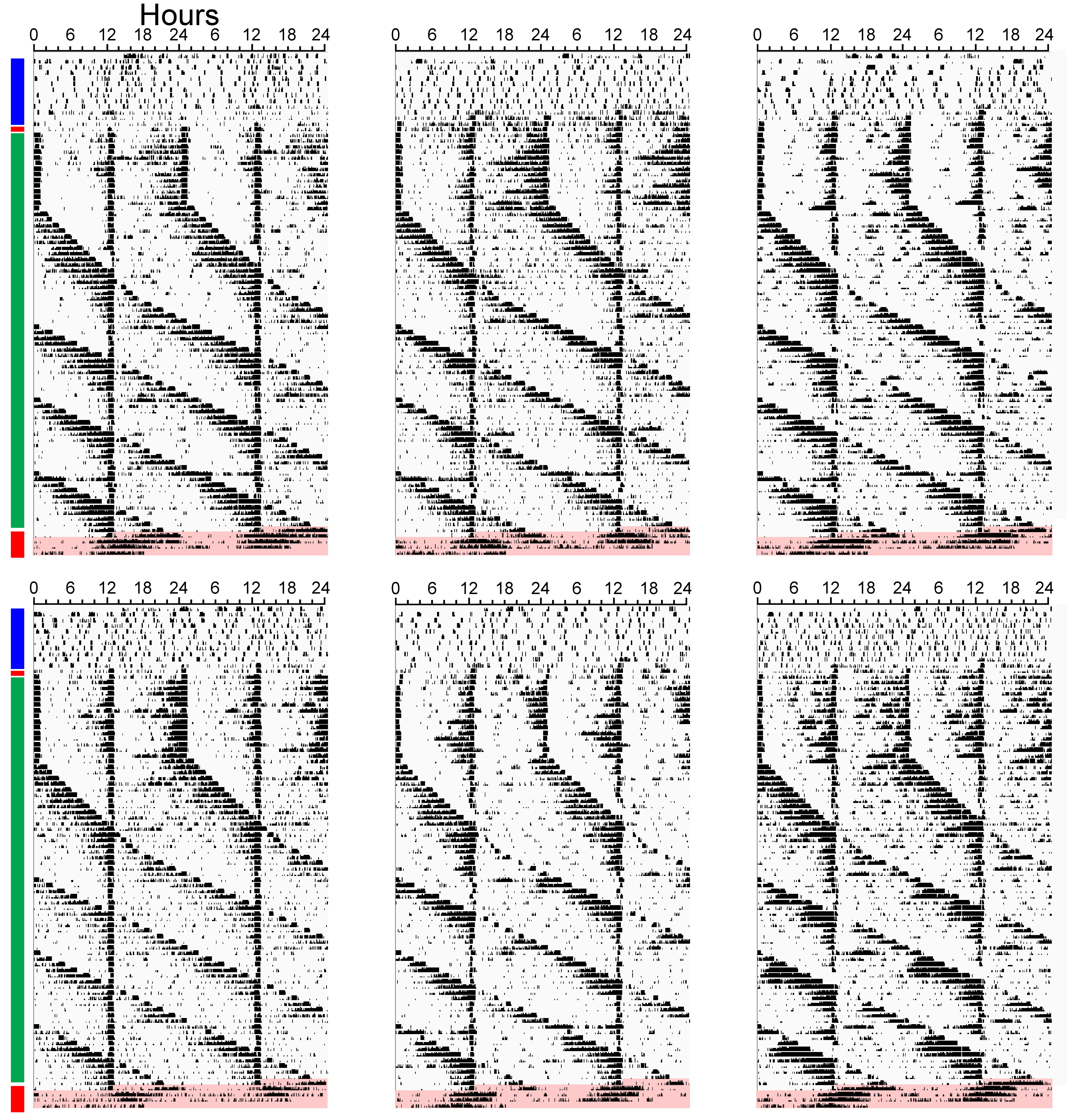


**Supplementary Figure S13.**

Periodicity of FAA components during 3-4 day food deprivation tests in constant dark (FD-DD) following the 2-meal T cycle feeding schedule (T24 in red, T26 in blue). Periodicity was measured by estimated the center of each FAA bout and then fitting a regression line to onsets on consecutive days. Group means are provided + standard deviations.


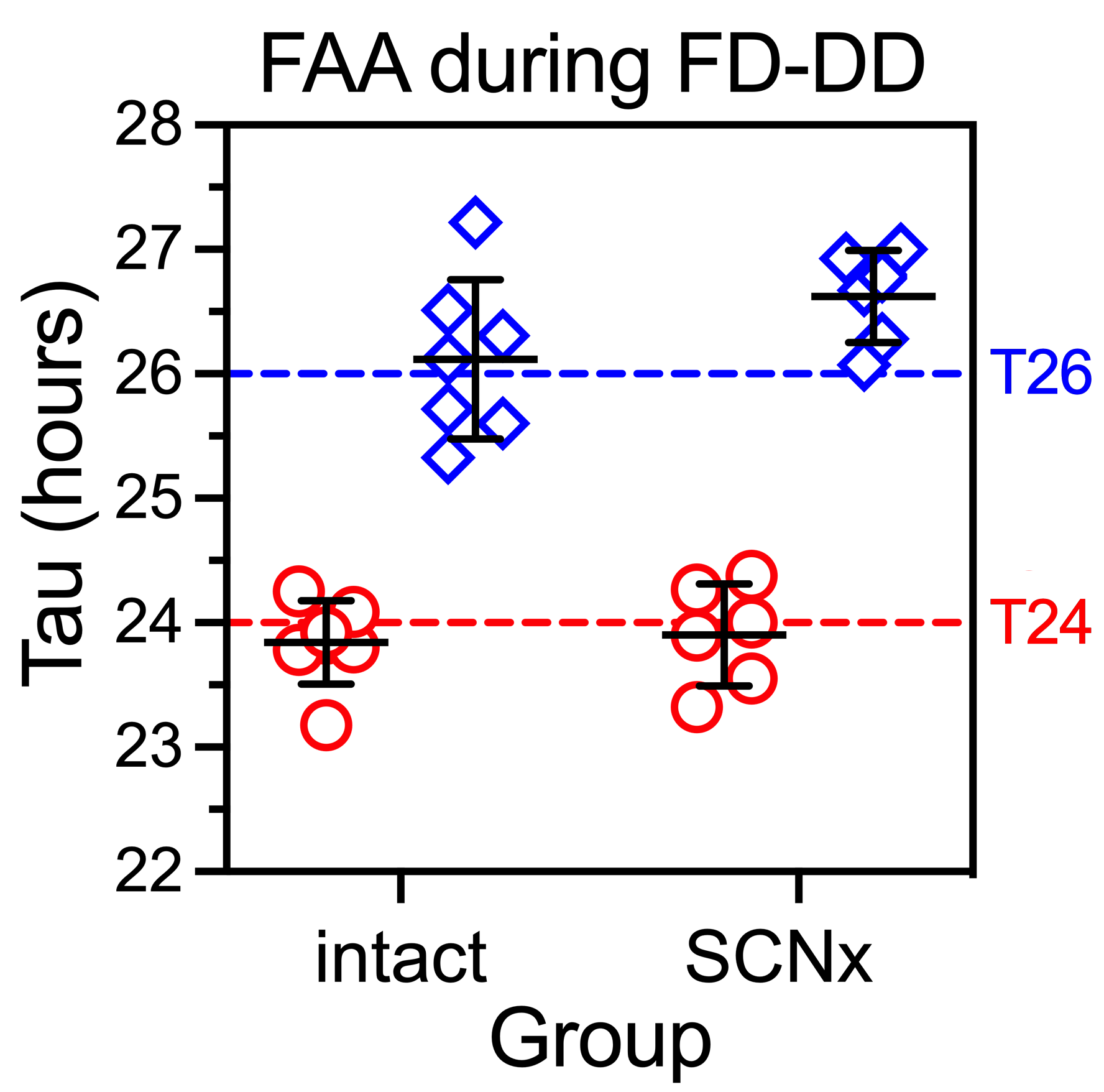

Supplement: Supplementary file 1 — Supplementary Figures. [file 41598_2022_13242_MOESM1_ESM.docx]
